# A diagnostic biomarker profile for fibromyalgia syndrome based on an NMR metabolomics study of selected patients and controls

**DOI:** 10.1186/s12883-017-0863-9

**Published:** 2017-05-11

**Authors:** Bontle G. Malatji, Helgard Meyer, Shayne Mason, Udo F.H. Engelke, Ron A. Wevers, Mari van Reenen, Carolus J. Reinecke

**Affiliations:** 1 Centre for Human Metabolomics, Faculty of Natural Sciences, North-West University (Potchefstroom Campus), Private Bag X6001, Potchefstroom, South Africa; 2 Department of Family Medicine, Kalafong Hospital, University of Pretoria, Private Bag X396, Pretoria, South Africa; 3 Translational Metabolic Laboratory, Department of Laboratory Medicine, Radboud University Nijmegen Medical Centre, PO Box 9101, 6500 HB, Nijmegen, The Netherlands

**Keywords:** Fibromyalgia syndrome, Proton nuclear magnetic resonance (^1^H–NMR) spectroscopy, Metabolomics, Metabolite markers, Pain

## Abstract

**Background:**

Fibromyalgia syndrome (FMS) is a chronic pain syndrome. A plausible pathogenesis of the disease is uncertain and the pursuit of measurable biomarkers for objective identification of affected individuals is a continuing endeavour in FMS research. Our objective was to perform an explorative metabolomics study (1) to elucidate the global urinary metabolite profile of patients suffering from FMS, and (2) to explore the potential of this metabolite information to augment existing medical practice in diagnosing the disease.

**Methods:**

We selected patients with a medical history of persistent FMS (*n* = 18), who described their recent state of the disease through the Fibromyalgia Impact Questionnaire (FIQR) and an in-house clinical questionnaire (IHCQ). Three control groups were used: first-generation family members of the patients (*n* = 11), age-related individuals without any indications of FMS or related conditions (*n* = 10), and healthy young (18–22 years) individuals (*n* = 20). All subjects were female and the biofluid under investigation was urine. Correlation analysis of the FIQR showed the FMS patients represented a well-defined disease group for this metabolomics study. Spectral analyses of urine were conducted using a 500 MHz ^1^H nuclear magnetic resonance (NMR) spectrometer; data processing and analyses were performed using Matlab, R, SPSS and SAS software.

**Results and discussion:**

Unsupervised and supervised multivariate analyses distinguished all three control groups and the FMS patients, and significant increases in metabolites related to the gut microbiome (hippuric, succinic and lactic acids) were observed. We have developed an algorithm for the diagnosis of FMS consisting of three metabolites — succinic acid, taurine and creatine — that have a good level of diagnostic accuracy (Receiver Operating Characteristic (ROC) analysis — area under the curve 90%) and on the pain and fatigue symptoms for the selected FMS patient group.

**Conclusion:**

Our data and comparative analyses indicated an altered metabolic profile of patients with FMS, analytically detectable within their urine. Validation studies may substantiate urinary metabolites to supplement information from medical assessment, tender-point measurements and FIQR questionnaires for an improved objective diagnosis of FMS.

**Electronic supplementary material:**

The online version of this article (doi:10.1186/s12883-017-0863-9) contains supplementary material, which is available to authorized users.

## Background

Fibromyalgia syndrome (FMS) is a common chronic pain syndrome characterized by widespread musculo-skeletal pain and associated with multiple other symptoms such as cognitive impairment, disrupted sleep and chronic fatigue. The American College of Rheumtology (ACR) first published criteria for FMS in 1990 [1] which emphasized chronic widespread musculo-skeletal pain (including pain in the axial skeleton) in the presence of pain on at least 11 of 18 specified tender point sites with digital palpation of 4 kg/cm2.

The 2010 ACR updated criteria for FMS [2] are applied in a 2-part, self-administered questionnaire and do not require a tender point assessment. The first part assesses the presence of pain at 19 sites on a body diagram (widespread pain index) and part 2 measures the symptom severity score (0–3) of 3 core symptoms (insomnia, fatigue and cognitive impairment) and an average score (0–3) for additional somatic symptoms. FMS is the most common cause of widespread or generalized musculo-skeletal pain and affects 2–8% of the adult population with the highest prevalence in women between 30 and 55 years. [3, 4].

FMS is currently viewed as a central sensitivity syndrome associated with abnormal pain processing. It is regarded as a “pain amplification syndrome” associated with increased sensitivity of the nervous system and decreased anti-nociception which results in the clinical phenomena of hyperalgesia and allodynia. Dysfunction in central mono-aminergic neurotransmission which involves serotonin, norepinephrine, nerve growth factor, substance P and others have been implicated in the patho-physiology of FMS. [5–8] FMS patients often have associated comorbidities such as irritable bowel syndrome, interstitial cystitis and mood disorders [9, 10].

In the absence of an objective biomarker, the diagnosis of FMS is based on a comprehensive clinical assessment. Before 2010, the diagnosis was principally based on the 1990 ACR criteria of widespread pain (including in the axial skeleton) > 3 months and at least 11 painful “tender points” with digital palpation. Although the 2010 ACR criteria do not include a “tender point” count, a musculo-skeletal clinical examination remains mandatory, to exclude other couses of widespread pain and also to identify peripheral pain generators e.g. myofascial trigger points. Selective use of laboratory testing is used to exclude other causes of widespread pain such as polymyalgia rheumatica and hypothyroidism.

The pursuit of specific and measurable biomarkers that may assist in objectively identifying susceptible individuals, confirming disease diagnosis and facilitating treatment, is a continuing endeavour in FMS research. The development of high-throughput metabolic profiling and the study of the metabolome have proven to be particularly applicable in neurological research where small molecules are key in neurochemical metabolism and in performing a role as neurotransmitters, signalling modulators and osmolytes. It is now generally anticipated that metabolomics profiling methods, linked to systems biology approaches, will emerge with well-defined metabolic phenotypes, enhancing the understanding of brain metabolism in health and disease. Recently, a few metabolomics studies have been reported on fibromyalgia, potentially disclosing novel insights into metabolic perturbations in the brain that go brain metabolic homeostasis beyond alterations of neurotransmission variations associated with neurological disorders [11].

In a pilot study, presented only as a poster at an Annual Meeting of the Rheumatologic Society of the UK [12], Richards and co-workers (2001) reported that muscle metabolites detected in the urine of fibromyalgia patients may suggest a prevailing muscle damage. Although not by definition a metabolomics study, their targeted metabolite analysis of urine by nuclear magnetic resonance (NMR) spectroscopy revealed significant levels of creatine in FMS patients and elevated (t-test *p* < 0.05) urinary excretion of choline, taurine, citrate and trimethylamine N-oxide (TMAO) relative to matched controls.

The first metabolomics study on FMS, reported in 2013 [13], used 50 μl blood samples collected on blood spot cards (Whatman 903 Protein Saver Snap Apart Card, GE Healthcare, Westborough, MA, USA) from patients diagnosed with FMS (*n* = 14), osteoarthritis (OA; *n* = 15) and rheumatoid arthritis (RA; *n* = 12). Samples were dried and then transported to the laboratory for mid-infrared micro-spectroscopy (IRMS) and other analyses. The RA and OA groups appeared to be metabolically similar, but different from the metabolite profile of FMS. The IRMS approach did not conclusively identify the metabolites responsible for the diagnostic spectral differentiation, although changes in tryptophan catabolism seemed to be involved.

Another metabolomics approach to FMS involved liquid chromatography/quadrupole–time-of-flight/mass spectrometry (LC/Q-TOF/MS) with multivariate statistical analysis aimed at discriminating FMS patients (*n* = 22) and controls (*n* = 21) from blood plasma analysis [14]. Lysophosphocholine (lysoPCs), phosphocholine and ceramide lipids dominated the metabolite profile. The metabolites that discriminated the most between FMS patients and controls were identified as 1-tetradecanoyl-sn-glycero-3-phosphocholine [PC(14:0/0:0)] and 1-hexadecanoyl-sn-glycero-3-phosphocholine [PC(16:0/0:0)] — suggesting that lysoPCs may be potential biomarkers for FMS.

In addition to these metabolomics findings, a recent review on biomarkers of FMS included contributions from genetic and proteomic studies [15]. Although genetic factors have been shown to influence predisposition to FMS, no specific genes have been confirmed as being involved in this disease [16]. The review also listed several proteins of the immune response, cytoskeleton remodelling and the inflammatory process in FMS. Their role in FMS, however, is still controversial.

Thus the availability of biomarkers for unequivocal and objective diagnosis of FMS remains elusive in clinical practice. Yet, metabolites identified as being involved in the aetiology and pathogenesis of FMS could meanwhile contribute to insights into various presentations of FMS and provide ancillary diagnostic testing criteria to complement general diagnostic procedures. We thus present here the outcomes of a ^1^H NMR metabolomics study on FMS. All experimental subjects were females and provided urine samples for the study. The investigation was designed as an untargeted approach and revealed metabolite information with predictive potential to discriminate between FMS patients and healthy young controls. The outcomes thus underscore the versatility of metabolomics to provide insights into disease pathophysiology, furthering potential novel approaches to supplement existing protocols proposed for the practising clinician to assess FMS and monitor its treatment [17].

## Methods

### Experimental subjects, physical characteristics, symptoms and clinical profiles

All the patients that were included in this study were previously diagnosed with FMS by the same specialist pain clinician from his chronic pain practice in Pretoria. This practice manages the full spectrum of chronic pain disorders, with a special interest in FMS and related pain disorders. The diagnosis was based on a comprehensive clinical assessment using the 1990 criteria. All patients in the study were confirmed with FMS before 2010 and all were on a comprehensive evidence-based management programme according to international guidelines. They were only included if they continued to complain of widespread musculo-skeletal pain (including in the axial skeleton) in the presence of >11 painful tender points with musculo-skeletal assessment.

Informed consent was obtained from all the participants in this study by means of a voluntarily completed consent form; ethical approval for the study was obtained as specified under Declarations. All participants in the study were female and the sample material investigated was urine. The experimental subjects consisted of one FMS patient group (Group 1) and three control groups (Groups 2 to 4). Clinical description and serum and urine sample collection on all experimental groups commenced from 2009 to 2011. Case definition and selection for the eventual study was done by clinical and scientific group of co-workers in 2010. Following scrutinizing of the records of patients with a medical history of FMS, a group of 18 FMS patients eventually selected based on the above selection criteria as well as after excluding outliers based on statistical analysis [(S5 in the Additional file 1: Supplementary Information (SI)].

The socio-demographic, tender point and myofascial pain experience, awareness of gastro-intestinal symptoms, pain-specific medication and levels of emotional experience associated with FMS for the 18 patients was obtained through the FIQR [17] and the IHCQ. The questionnaires are presented in Table S1 and the response to the IHCQ are summarised in Table S2 of the SI. The IHCQ included 18 items that could be extended to a total of 30 sub-items. The questionnaire provided socio-demographic information on the patients (3 items), personal clinical experience of pain and their FMS condition (5 items) and use of medication against pain (2 questions), emotional experience (7 questions) and digestive functioning (1 item). The urine samples were provided by the patients prior to application of pressure to the tender-points (TPs). For a total of 16 of these FMS patients a complete set of data was available for the comparative analysis of the FIQR and metabolomics data, as some information on some patients had to be excluded because the data were incomplete. Some degree of comorbidity of conditions that overlap with FMS (e.g. chronic fatigue syndrome) could not be excluded, as the mean level of energy in the FIQR was rated at 7,0 and according to responses to the IHCQ, 94% of the patients experienced sleep disturbances and did not awoke refreshed. The responses to an experience of mood disturbances (58% answered “Yes”) and anxiety (52% answered “Yes”) for the FMS patients as a group were moderate. Responses on depression was inconsistent (mean FIQR-score = 5.1 with 84% “Yes” answers on the IHCQ) but 88% indicated discomforts with their gastrointestinal functions (Indicated as Irritable Bowel Syndrome (IBS) in the IHCQ). These scores were accepted as indications of the mental and physical profile of the FMS patient group and were not further clinically verified.

Three control groups were used: (1) a group of 11 subjects that were first-degree relatives of the patients, meaning that they were a mother, sister or daughter relation (Group 2: CF); (2) a group of 10 unrelated subjects, selected by physicians and defined as unrelated and age matched controls to the patients (Group 3: CO); (3) a control group of young and healthy individuals, comprising 20 randomly selected students (aged 18–22 years) of North-West University (NWU) (Group 4: CN). All individuals in the control groups showed no indications of FMS or related conditions and was not required to complete the FIQR or IHCQ.

This investigation used availability sampling on the clinically selected FMS patients and controls (CO, CF and CN). However, statistical analyses indicated that the sample sizes provided sufficient power to detect large effects at a univariate level.in the FMS and CN comparison.

### Sample preparation and ^1^H NMR analysis

Spectral analyses were conducted according to the protocol at the NMR facility of the Translational Metabolic Laboratory at Radboud University Medical Centre in Nijmegen, the Netherlands [18, 19]. The urine samples were collected in South Africa, stored at –80 °C and transported to the Netherlands before being thawed at room temperature for analysis. A 1 ml volume of each sample was centrifuged at 3000 rpm for 10 min to remove any sediments or debris. A 70 μl volume of a deuterated solution containing 20.2 mM of trimethyl-2,2,3,3-tetradeuteropropionic acid (TSP, sodium salt; Sigma Aldrich) was added to 700 μl of the supernatant and vortexed. This internal standard (IS) solution served to lock the signal during analysis and to provide a chemical shift reference of δ = 0.00. The sample was then acidified to pH 2.5 ± 0.05, with 37% concentrated hydrochloric acid (HCl). A 650 μl aliquot of the acidified sample was then transferred to a 5 mm NMR tube (Wilmad Royal Imperial; Wilmad LabGlass, USA) and analysed on a 500 MHz Bruker Avance spectrometer (Bruker Analytische Messtechnik, Karlsruhe, Germany) (pulse angle 90^o^, delay time 4 s, number of scans 256, temperature 298 K). Water suppression was achieved by using gated irradiation focused on the water frequency. All samples were automatically shimmed prior to acquisition of data, using topshim from Bruker BioSpin. The resultant raw spectral data, in the form of free induction decay, were Fourier transformed. These transformed spectra were then manually corrected for phase and baseline. All the samples were normalized with reference to the creatinine CH_3_ peak at 3.13 ppm. We opted for two methods of spectral analysis. The first method entailed equidistant binning [20] using a bin width of 0.02 ppm applied to the selected region of 0.5–10 ppm, which gave a total of 461 integrated units per NMR spectrum, excluding the water region, for each individual of the four experimental groups. The second method entailed variable-sized binning. The equal-binning procedure masks subtle chemical shift differences, hides potentially significant changes of low-intensity peaks and incurs the risk of splitting peaks or spectral features between bins [21]. To avoid these problems we also used variable bin sizes in areas of peaks above the noise level, preventing peak division between multiple bins. This approach was specifically applied for the identification and quantification of discernible and important known metabolites, generating data for univariate analysis.

### Data and statistical analysis

The original normalized spectral data (presented in Additional file 1 as Table S4 in S1 – Supplementary information (SI) or Additional file 2 – Raw data matrix) were pre-processed by performing log transformation and auto-scaling. Outliers were detected through Hotelling’s T^2^ and PCA scores (using a 90% confidence region) analysis and resulted in the exclusion of 4 outliers from further analysis. Univariate statistical analyses, specifically the Mann–Whitney test *p*-values (MW) and associated effect sizes (ES), were generated for each feature. Multivariate analyses were performed using cluster analysis (Euclidean distance and Ward linkage) principal components analysis (PCA) and partial least squares discriminant analysis (PLS-DA), using a 90% confidence interval (CI). Data processing and analyses were performed using Matlab (MATLAB with Statistics and PLS Toolbox Release 2012b, The MathWorks, Inc., Natick, MA, USA); R (R version 3.2.3 downloaded from https://www.R-project.org with the corrplot package downloaded from https://cran.r-project.org/web/packages/corrplot); the SPSS software package (SPSS Inc. (2015). IBM SPSS Statistics Version 22, Release 22.0.0, © IBM Corporation and its licensors - http://www-01.ibm.com/software/analytics/spss/) and SAS (SAS Institute Inc. 2016 The SAS System for Windows Release 9.4 TS Level 1 M3, SAS Institute Inc., Cary, NC, USA). A table containing all discriminant information, i.e. the power and VIP values as generated from the PCA and PLS-DA analyses, respectively, as well as the ES and MW *p*-values, was constructed.

We did not test for a normal distribution of the data, given the small number of cases and used Pearson’s r and Spearman’s rho to assess correlations, analysed through SPSS version 12.0 (SPSS, Inc., Chicago, IL). All tests were one-tailed, given the positive fold changes (FC) observed for all metabolites.

## Results

### Characteristics of the FMS patient group

The age profile of the patients concurs with the general agreement of FMS being uncommon in young subjects (<25–30 years), increasing with age towards the prevalence peak in middle-aged individuals, and then declines [3, 4]. According to the feedback, 88% of our patients had stable relationships with a male partner, 89% had one form or another of day-filling or employment activities, and their emotional experience was not severely affected by their disease. The pain experience and medication used resembled that generally prescribed for FMS. The mean scores and ranges of the 21 FIQR questions obtained for our patient group and those (designated as the reference group) used for the standardization of the questionnaire [17] are compared in Table S3.

**Fig. 1:**
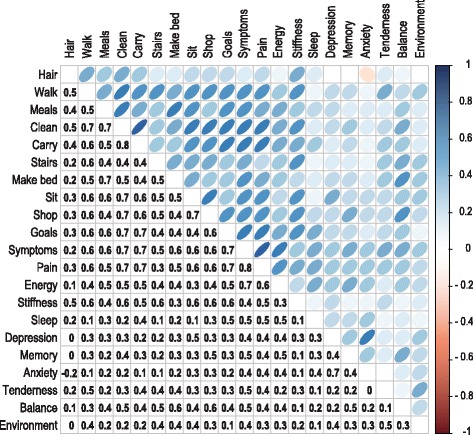
Correlation matrix for all items on the FIQR questionnaire. Full details on the data analysis are included in the SI

To characterize further the relationship between questions or variables making up the FIQR questionnaire, we calculated Kendall’s tau correlation coefficients for the FMS patient group (Fig. 1). The correlation coefficients along with their associated significance levels are indicated in Table S3. The highest correlation (*r* = 0.817) was indicated for the relationship between pain and the symptoms for FMS. The function domain contains 9 physical functioning items related to the ability to perform relatively demanding but regular daily muscle tasks. Apart from the low score for ‘brushing hair’, all remaining items showed high correlation coefficients among each other, ranging from 0.399 to 0.778. These high values collectively substantiate the major signs and symptoms experienced by the FMS patients. The ‘overall impact’ domain contained 2 items that asked about the number of days individuals felt well (could reach their goals) and the corresponding number they were unable to work because of FMS symptoms. These again showed high correlation coefficients, ranging from 0.421 to 0.686, with the 8 items in the functional domain indicating the underlying negative impact of the FMS symptoms on the daily routine of the FMS patients. The symptoms domain contained 9 items on which patients had to rate work and physiological, psychological and environmental difficulties related to FMS. Lower correlations, ranging from 0.076 to 0.499 (mean = 0.25), were found between the 8 functional items and sleep patterns, memory, anxiety and depression, indicating little overlap within the patient group with other FMS-related conditions. Taking everything into account, we conclude the FMS patients represent a well-defined group for this explorative metabolomics study.

### Data generation and case selection

**Fig. 2:**
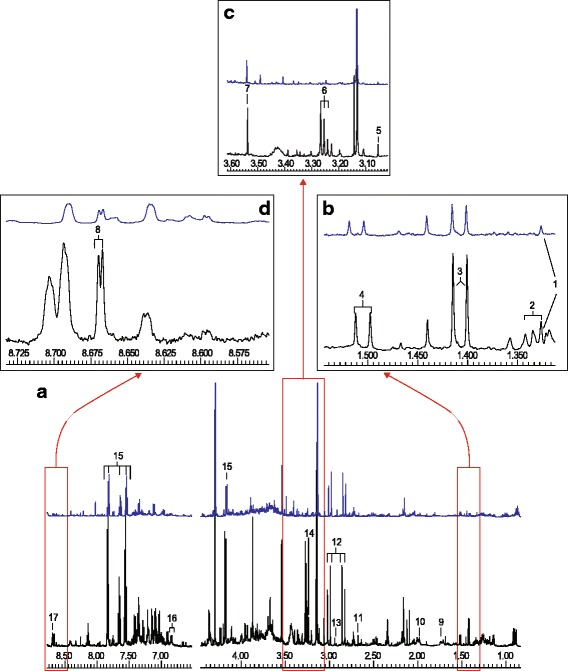
Representative spectra from one FMS patient (**b**, *black*) and one young control subject (**a**, *blue*), both scaled according to the creatinine CH3 peak at 3.13 ppm. Expanded regions (**c**-**e**), framed in *red* in the spectra, are the regions where variables important in projection (VIP) through the supervised PLS-DA are located. The labelled metabolites with their chemical shift (in ppm) and multiplicity, respectively, indicated in brackets are given numerically as follows: 1, 3-hydroxyisovaleric acid (1.33 s); 2, threonine (1.33 d); 3, lactic acid (1.41 d); 4, alanine (1.50 d); 5, creatine (3.05 s); 6, taurine (3.25 t, 3.42 s – *broad line*); 7, trimethylamineN-oxide (TMAO) (3.54 s); 8, histidine (8.68 d); 9, 2-hydroxyisobutyric acid (1.44 s); 10, N-acetyl-X (2.03 s); 11, succinic acid (2.67 s); 12, citric acid (2.91 AB); 13, N,N-dimethylglycine (2.93 s); 14, carnitine (3.22 s); 15, hippuric acid (4.18 d, 7.55 t, 7.64 t, 7.83 d); 16, tyrosine (6.89 d); 17, histamine (8.70 d); 18, creatinine (3.13 s, 4.29 s)

Representative scaled NMR spectra from an FMS patient and from the young control group (CN) is shown in Fig. 2 to illustrate some of the discernible qualitative NMR differences observed in these selected examples. Close inspection of the spectra indicates that there were no immediately discernible qualitative differences between the two representative examples, suggesting that FMS is not associated with distinctive metabolic aberrations, as otherwise observed in monogenetic disorders such as inborn errors of metabolism. Using the equal-bins spectral data, case reduction was first applied to all four experimental groups (Additional file 1: Figure S2). Four outliers were identified using a 95% confidence region in a Hotelling’s T^2^ test in conjunction with the respective PCA score plots with 90% confidence regions. Cases that were identified as outliers by either method were removed. The outliers were: group 1 (FMS patients) – one outlier; group 2 (CF; family controls) – two outliers; group 3 (CO; matched controls) – no outliers; group 4 (CN; young controls) – one outlier.

### Group characteristics

Supposed changes in metabolite profiles from the FMS patients and the three control groups (excluding outliers) were established through three multivariate approaches: unsupervised Euclidian and Ward hierarchical cluster analyses presented as dendrograms, unsupervised PCA, and supervised PLS-DA models, applied to the original 461 ^1^H NMR profiled bins for the four experimental groups.

**Fig. 3:**
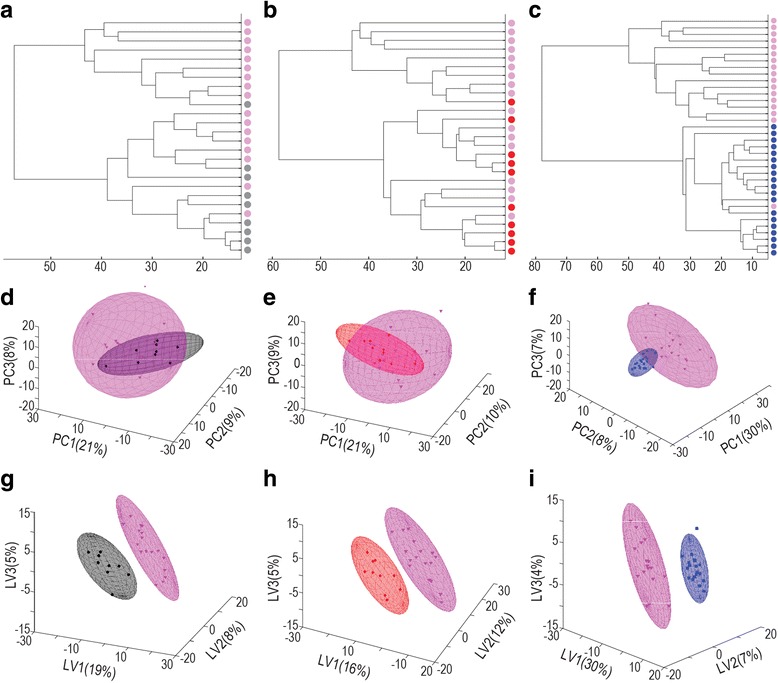
Group separation between experimental groups through cluster and multivariate analysis based on equidistant binning data. (**a–c**): Dendrograms from cluster analysis are shown for the CF (**a**), CO (**b**) and CN (**c**) groups relative to FMS patients. Cases from the FMS patients are shown as *pink dots*, CF as *black*, CO as *red* and CF controls as *blue*. (**d–f**): PCA indicating the group separation between the FMS patients and CF (**d**), CO (**e**), and CN (**f**) groups respectively, with areas using the same colour code as the *dots* in the dendrograms. (**g–i**): PLS-DA indicating the separation between the FMS patients and CF (**g**), CO (**h**), and CN (**i**) groups respectively, with areas using the same colour code as in the PCA

Figure 3 shows the group separations based on the unsupervised cluster analysis, indicating the perceived closeness of spectral data encapsulated in the NMR bins. The main clusters formed between the CF family members group (Fig. 3a) and the CO age-matched group (Fig. 3b) relative to the FMS patients are heterogeneous in terms of case distribution. In contrast, two well-defined clusters were formed between the FMS patients and CN young controls (Fig. 3c), suggesting distinct differences in the spectral fingerprints between these two groups.

Next, group separations based on unsupervised PCA and supervised PLS-DA were performed. The data were log transformed and auto-scaled. The PCA between the CF family members (Fig. 3d), CO matched controls (Fig. 3e) and FMS patients complemented results from the cluster analyses. A complete separation was obtained between all three control groups and the FMS patients (Fig. 3f–i) through supervised PLS-DA. Evaluation of the PLS-DA model shown in Fig. 3i (FMS vs CN) was performed by calculating the goodness-of-fit (R^2^) and predictive ability (Q^2^) parameters. These metrics confirmed the complete separation between the FMS and CN young control groups, with good model fit (R^2^ = 0. 96), however this model may not generalize well (Q^2^ = 0.29).

From the equal binning analysis it is evident that there are bins or combinations of bins that can discriminate between our patient and control groups. However, since it is not clinically practical to measure bins, we did not investigate this data further. Instead, the metabolites potentially responsible for the separation of the FMS patients and the CN young controls were subsequently identified by analyzing variable bins from the NMR spectra and converting these measures to concentration values of the identified metabolites.

### Metabolite profile of the FMS patient group

Twenty-one metabolites could be identified and quantified from the NMR spectra. From this list we selected twenty endogenous metabolites (listed in Table 1), and also included 2-hydroxyisobutyric acid of exogenous origin [22], with high VIP, ES and ES values, despite being present in low concentrations. The endogenous metabolites include seven amino acids (tyrosine, leucine, valine, histidine, alanine, threonine and lysine), seven metabolites directly or indirectly associated with energy metabolism (lactic acid, succinic acid, citric acid, 3-hydroxyisovaleric acid, creatine, carnitine and formic acid), three osmolytes (taurine, TMAO and dimethylglycine), a major mammalian detoxification product (hippuric acid), histamine and an N-acetyl-derivative. The N-acetyl-derivative showed a singlet at 2.03 ppm, possibly indicative of an N-acetyl group. One-dimensional spectral data suggested that aspartic acid (multiplet at 4.70 ppm) could be the moiety linked to the N-acetyl group, which, however, could not be substantiated as N-acetyl-aspartic acid by two-dimensional NMR spectral analysis Additional file 1: Figure S4). We thus designated the variable as an N-acetyl derivative (N-acetyl-X).

**Table 1:** Univariate, multivariate and descriptive statistics for the 20 bins, comparing FMS and CN

Variable	CS and M[Ps]	VIP	Mann-Whitney		Fold Change	Mean		StDev	
		3 LV	*p*-value	Effect size		CN	FMS	CN	FMS
2-Hydroxyisobutyric acid	1.44 s [CH3]	6.26	0.0001	0.72	−1.56	0.01	0.02	0.0	0.00
Succinic acid	2.66 s [(CH2)2]	0.25	0.0001	0.61	−1.63	0.02	0.03	0.01	0.01
Taurine	3.25 t [CH2]	5.21	0.0007	0.52	−2.29	0.20	0.45	0.05	0.57
Tyrosine	6.89 dd [(CH)2]	0.37	0.0029	0.45	−1.70	0.03	0.06	0.03	0.06
Lactic acid	1.41 d [CH3]	2.83	0.0044	0.42	−1.81	0.06	0.11	0.03	0.07
Creatine	3.05 s [CH3]	4.40	0.0053	0.41	−2.08	0.05	0.09	0.04	0.08
TMAO	3.54 s [(CH3)3]	2.21	0.0062	0.41	−2.10	0.06	0.14	0.06	0.23
Dimethylglycine	2.93 s [(CH3)2]	0.00	0.0127	0.36	−1.29	0.01	0.01	0.00	0.00
Leucine	0.95 t [(CH3)2]	0.00	0.0136	0.36	−1.11	0.01	0.02	0.00	0.00
Formic acid	8.25 s [CH]	0.01	0.0361	0.29	−1.15	0.03	0.03	0.01	0.01
Valine	1.04 d [CH3]	0.00	0.0436	0.28	−1.24	0.01	0.01	0.00	0.00
Histamine	8.70 d [CH]	0.08	0.0436	0.28	−1.29	0.06	0.07	0.06	0.05
N-acetyl-X	2.03 s [CH3]	0.02	0.0464	0.27	−1.28	0.01	0.02	0.00	0.01
Lysine	1.73 m [CH2]	0.61	0.0739	0.23	−1.03	0.11	0.12	0.03	0.06
Hippuric acid	4.18 d [CH2]	1.61	0.0966	0.21	−1.55	0.22	0.35	0.10	0.24
Citric acid	2.89 AB [(CH)4]	1.36	0.1070	0.20	−1.21	0.39	0.47	0.16	0.17
Alanine	1.51 d [CH3]	0.13	0.1785	0.15	−1.16	0.06	0.07	0.02	0.03
Histidine	8.68 d [CH]	0.85	0.1942	0.14	1.19	0.07	0.06	0.04	0.04
Carnitine	3.22 s [(CH3)3]	0.02	0.2107	0.13	−1.24	0.02	0.02	0.01	0.01
Threonine	1.33 d [CH3]	0.04	0.2648	0.10	−1.28	0.03	0.04	0.01	0.04
3-Hydroxyisovaleric acid	1.33 s [(CH3)2]	0.00	0.4942	0.00	−1.02	0.00	0.00	0.00	0.00

**Fig. 4:**
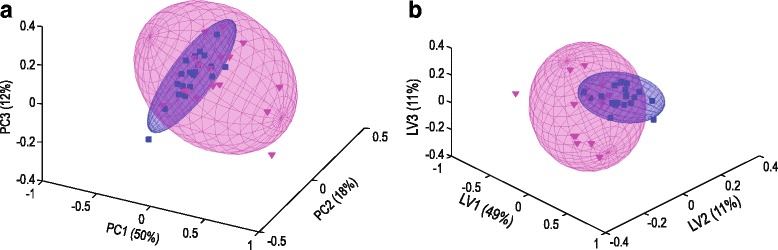
PCA (**a**) and PLS-DA (**b**) for the FMS patients relative to the young controls, based on the quantified 20 metabolites

We subsequently performed multivariate (log and centred concentration values) and univariate (unscaled concentration values) analyses on the reduced bins (endogenous metabolites, converted to their respective metabolite concentrations) to refine our identification of the key variables that discriminate between the FMS patients and the controls. All cases were retained for this analysis as none were identified as outliers based on the concentrations. Multivariate PCA (Fig. 4a) and PLS-DA (Fig. 4b) both indicated that the 20 metabolites contained information that differentiates, but did not separate, the FMS patients from the young controls. Model performance was evaluated using the goodness of fit (R^2^) and goodness of prediction (Q^2^) parameters, which were R^2^(X) = 0.52 and Q^2^(Y) = 0.05, respectively, indicating a reasonable (R^2^) but not necessarily reproducible (Q^2^) fit between the variation in the data and the components (quantified metabolites) comprising the model for the present FMS group. It thus appears that some metabolites below the sensitivity range for quantification from the NMR spectra might be required for reproducibility (Q^2^) and for further differentiation between the FMS patients and young controls.

**Fig. 5:**
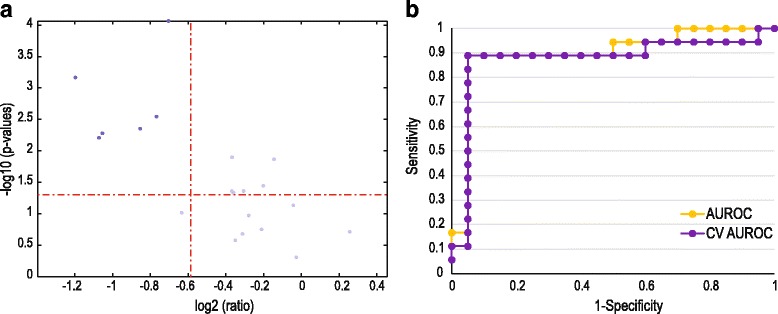
Statistical assessments of three metabolites indicative of FMS: (**a**) Volcano plot mapped by the scaled fold change and *p*-values for the 20 metabolites observed for FMS patients and young controls. Metabolites with high FC and significant *p*-values among patients are indicated by *black squares*. (**b**) ROC analyses for discriminating FMS patients from controls (AUROC) as well as leave-one-out crossvalidated ROC analysis (CV AUROC). The discriminator consisted of the three informative metabolites (succinic acid, taurine and creatine) identified by multivariate, univariate and metabolic pathway analyses

Univariate analyses using Mann–Whitney *p*-values and fold changes, as summarized in a volcano plot (Fig. 5a), point to important substances that cause group differentiation. The outcome of this analysis of the data set of 20 variables is presented in Fig. 5a, indicating which large-magnitude changes (fold change: |log_2_ FC| ˃ 1.5) are also statistically significant (Mann–Whitney test: *p* < 0.05). Six informative metabolites complied with these measures, with their respective VIP values shown in brackets: succinic acid (0.246), taurine (5.214), tyrosine (0.365), lactic acid (2.832), creatine (4.402) and trimethylamine N-oxide (TMAO; 2.209).

### Important endogenous metabolites in the FMS patient group

A summary of the results for the univariate and multivariate statistical analyses is presented in Table 1, nine of which could be related to physiological functions that could be related to FMS.

The neurological functions of succinic acid, tyrosine and lactic acid are well known: the aerobic mitochondrial energy regeneration function, a precursor for neurotransmitters and a key metabolite in the astrocyte-neuron lactate shuttle [23], respectively. Taurine is an abundant β-amino acid in the mammalian brain [24] and has been shown to be a neurotransmitter in the substantia nigra (SN). It has been suggested from micro-dialysis experiments on Sprague-Dawley rats that osmoregulation of the nonsynaptic taurine pool of the SN could influence the nigral cell vulnerability, seen in the pathogenesis of Parkinson’s disease [25]. Likewise, nutritional studies [26] suggest that TMAO may be involved in diet-induced variations in the balance of several osmolytes, including betaine, choline, creatinine and creatine, whereas creatine has also been proposed as being involved in pain experienced in FMS [25]. Thus, we subsequently evaluated the potential diagnostic value of these six metabolites on FMS by means of a logistic regression analysis, as indicated below.

### Important exogenous metabolites in the FMS patient group

**Fig. 6:**
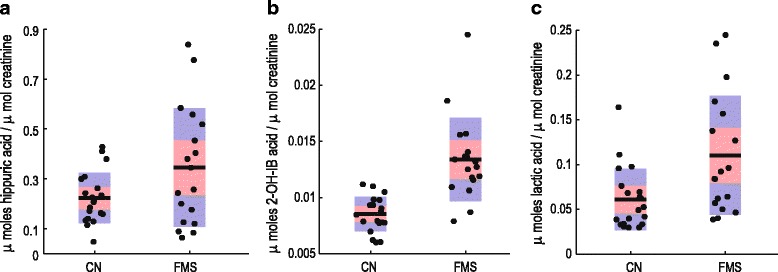
Graphs showing important urinary metabolites related to the gut microbiome. Indicated in the figure are: FMS patients relative to young controls for hippuric (**a**), 2-hydroxyisobutyric (**b**) and lactic (**c**) acids. Values for all individual cases are shown as *dots*, while the squared area represents the 95% confidence interval (*orange*) and 1 standard deviation (*blue*) of the mean (*red line*)

The pain intensity of patients with FMS has been reported to correlate with the degree of small intestinal bacterial overgrowth [9, 10]. This clinical observation may have pathogenetic relevance for FMS, because bacterial overgrowth leads to the exposure of immune cells to luminal antigens and consequent immune modulation. An untargeted NMR metabolomics study of celiac disease, a multifactorial immune-mediated enteropathy [27], suggested alterations of energy metabolism - a clinical characteristic in FMS - while urine data pointed to alterations of gut microbiota. At least three metabolites observed in the urine samples of our FMS patient group suggest perturbations in their gut metabolome (Fig. 6): (1) Hippuric acid is a normal and major component of urine and appear in humans as an increased excretory product from unnatural (detoxification) and natural (gastroesophageal reflux disease in children) sources. (2) 2-Hydroxyisobutyric acid, the most discriminatory variable between our FMS group and controls (VIP = 6.2 – Table 1), is an apparent catabolic from gut microbiotica and was shown to be statistically linked to *Faecalibacterium prausnitzii* [28] an important commensal bacterium of the human gut flora proposed to be an indicator of the dynamic basis of host–microbiome symbiosis. (3) Lactic acid is a key intermediate in many biochemical processes and is a measure of critical illness in patients with poor prognosis. It may be of endogenous (L-lactate) or exogenous (D-lactate) origin and we recently proposed that the determination of its enantiomers in infectious conditions may provide a basis for substantiating the clinical significance of disease markers [29]. The presence of these exogenous markers of gut origin provides further indications of the connectivity between disturbances in the gut microbial populations and the metabolic consequences of the altered microbial–mammalian metabolic balance influencing host disease, which will be discussed below in the context of FMS.

### A putative biosignature for FMS

A combination of three selection methods (Forward, Backward and Step-wise selection) was used to identify the best metabolite predictors. Instead of using one selection method, a combined approach was chosen since each method has its advantages and disadvantages [30, 31]. Although our aim was to explore a small set of highly discriminatory endogenous metabolites, we also investigated the potential of a combination of these metabolites to function as a biosignature for the FMS patient group. We followed a forced entry approach to evaluate the combination of metabolites. Table 2 lists the methods used as well as the preferred metabolite predictors selected from the six informative metabolites. The last model (Forced entry) entered succinic acid, taurine and creatine, and produced the best model based on −2 Log Likelihood (−2LL) from the present data. Table 2 also reports other model performance measures, but -2LL was used to select the best model as it gives an indication of the variation not explained in the data, and gave the lowest -2LL value compared to the other models. The Forced entry model was also well calibrated since the Hosmer Lemeshow (HL) statistic was not significant. The model fit is reported by using the Max Rescaled R-squared value and only the Forced entry model had a satisfactory value of above 0.6.

**Table 2:** Summary of logistic regression results for the six informative metabolites. The predictors used or selected by the logistic regression model are listed as *Predictors selected*. Other columns report the model fit results (Max Rescaled R-squared), the relative variance explained (−2LL), the calibration (HL *p*-value), and the classification ability (AUC and AUC (LOO CV)) of each model

Selection method	Predictors selected	-2LL	HL *p*-value	Max rescaled R-squared	AUC	AUC (LOO CV)
Forward	Creatine & succinic acid	36.15	0.0273	0.47	0.8917	0.8583
Backward	Taurine	40.16	0.6336	0.37	0.8056	0.7556
Stepwise	Succinic acid	40.87	0.5496	0.35	0.8583	0.8306
Forced Entry	Creatine; succinic acid & taurine	29.66	0.0932	0.60	0.8972	0.875

Finally, the classification ability of each model was assessed by using a Receiver Operating Characteristic (ROC) analysis to the data mentioned. The values of the area under the ROC curve (AUC) provide a measure of how well this combination could distinguish between the two groups. A value of AUC = 1 represents a perfect test, while a cursory guide for classifying the accuracy of a diagnostic test is given by: AUC = 0.90–1 (excellent, i.e. high sensitivity and high specificity); 0.80–0.90 (good); 0.70–0.80 (fair); 0.60–0.70 (poor); 0.50–0.60 (fail). To provide some indication of how well the model would potentially generalize, the last column in Table 2 reports the classification ability when one sample is left out repeatedly — in other words, based on a leave-one-out cross-validation strategy (AUC (LOO CV)). Again the Forced entry model performed the best (AUC = 90% [0.8972]; AUC (LOOCV) = 88% [0.8750]).

### Correlation between clinical and metabolic indicators

Pearson and Spearman correlation analysis was done to compare the bivariate relationships between responses to the FIQR and the three endogenous variables defining the biosignature of FMS. Specifically, correlations were assessed between the sum of all three FIQR domains as well as the sum of the functional, impact and symptoms domains and SUM-3, SUM-2, creatine, succinic acid and taurine. Finally we inspected the data for symptoms related to metabolism to be included in the bivariate correlation analysis. In this regard it should be noted: (1) The scores of the 21 questions of the FIQR corresponds to an average based on the subjective self-assessment of the FMS patients as used in the behavioural sciences (i.e., it is not empirically based). We therefore used the mean scores of fibromyalgia patients on the symptoms for experience of pain, low energy levels and tenderness to touch only as a directive to include these symptoms in the bivariate correlation analysis [30]. Their mean values did not differed in practice from the data of a reference group of the revised FIQR (Additional file 1: Figure S1). (2) The number of FMS cases is relatively small for assessment of normality in the data distribution. We therefore included the Pearson and Spearman correlations in Table 3, but used only the Spearman’s correlations for the interpretation of the bivariate correlation analyses, with guideline values for “small”(*r* ≥ 0.1), “medium” (*r* ≥ 0.3) and “large” (*r* ≥ 0.5) as operational convention for the correlation coefficients [32].

**Table 3:** Relationship between the clinical information of the FIQR and the components of the FMS biosignature

Bivariate components for the correlation analysis	Pearson correlation		Spearman correlation	
	Coeff. (*r*)	*p*-value^b^	Coeff. (*r*)	*p*-value
Correlations of the biosignature (SUM-3)^a^ with the FIQR domain categories				
SUM-3 vs Sum of 21 questions of the full FIQR	0.35	0.102	0.42	0.059
SUM-3 vs Sum of 9 questions of the functional domain	0.31	0.134	0.25	0.134
SUM-3 vs Sum of 2 questions of the impact domain	0.15	0.316	0.22	0.241
SUM-3 vs Sum of 10 questions of the symptoms domain	0.41	0.057	0.57	0.011*
Correlations of two components of the biosignature (SUM-2) with the FIQR domain categories				
SUM-2 vs Sum of 21 questions of the full FIQR	0.56	0.016*	0.53	0.021*
SUM-2 *vs*Sum of 9 questions of the functional domain	0.52	0.023*	0.41	0.008**
SUM-2 vs Sum of 2 questions of the impact domain	0.5	0.043*	0.51	0.039*
SUM-2 vs Sum of 10 questions of the symptoms domain	0.59	0.009**	0.57	0.011*
Correlations of components of the biosignature with the symptom of pain^c^				
SUM-3 vs pain experience	0.46	0.037*	0.64	0.004**
SUM-2 vs pain experience	0.52	0.02*	0.54	0.016*
Creatine vs pain experience	0.5	0.025*	0.5	0.024*
Succinic acid vs pain experience	0.08	0.384	0.18	0.249
Taurine vs pain experience	0.39	0.069	0.29	0.135
Correlations of components of the biosignature with the symptom of energy^d^				
SUM-3 vs energy loss	0.32	0.115	0.61	0.006**
SUM-2 vs energy loss	0.68	0.002**	0.72	0.001**
Creatine vs energy loss	0.65	0.003**	0.66	0.003**
Succinic acid vs energy loss	0.15	0.295	0.22	0.221
Taurine vs energy loss	0.22	0.204	0.14	0.307

^a^Biosignature: SUM-3 = creatine + succinic acid + taurine; SUM-2 = creatine + succinic acid

^b^Statistical significance: *significant at *p* ≤ 0.05, **significant at *p* ≤ 0.01

^c^Pain: No pain = 0; Unbearable pain = 10

^d^Energy: Lots of energy = 0; No energy = 10

The results shown indicate a medium and borderline significant relationship between the SUM-3 biosignature and the sum of the FIQR, with insignificant correlations for its functional and impact domains. Sum-3 and the symptoms domain showed a large and significant correlation. The relationship between SUM-2 and the sum of the FIQR and its three domains improved significantly. Taken together these results directs to a more meaningful relationship between the metabolites which comprise the biosignature and clinical symptoms related to biochemical perturbations in FMS. This impression is substantiated by the strong and significant relationship between SUM-3 and SUM-2 on the experience of pain (*p* = 0.004 and 0.016, respectively) and loss of energy (*p* = 0.006 and 0.001, respectively) in the FMS patients as a group. Notably this relationship is not shared by succinic acid (a metabolite from the Krebs cycle) and taurine (an osmolite), but a good and significant relationship was shown between creatine and the symptoms pain and energy (*p* = 0.024 and 0.003, respectively). The relationship between the biosignature components to tenderness to touch, the third clinical symptom evaluated, was statistically insignificant (not included in Table 2). All correlation coefficients were positive indicating that patients with high scores on the biosignature will likely also have high FIQR scores.

## Discussion

The results of this metabolomics study lead to three main discussion points – whether FMS presents with a unique global metabolic profile which characterizes this disease, whether metabolomics studies contributed to the advancement of an objective clinical diagnosis of FMS in patients so affected and on gut microbial–host metabolic perturbations in FMS.

As the overall health status of individuals is captured in their metabolic state, there exists a prevailing view that metabolomics results embody global biochemical changes in an individual due to a disease and neurological conditions [33], and supported by our results and of two other NMR metabolomics investigations. The first NMR metabolomics study evaluated the diagnostic accuracy of biomarker profiles in three neurological conditions: idiopathic intracranial hypertension, multiple sclerosis, and cerebrovascular disease relative to controls with either no or combined neurological diseases [34]. It appeared that the metabolomics investigation identified differences in metabolite profiles in patients suffering from these three conditions. A related conclusion was drawn from the second NMR metabolomics study of FMS [14]. Although a relatively small number of patients formed the experimental group, the metabolomics approach was successful in identifying distinct metabolic profiles for FMS patients relative to controls, supporting the concept that the Platelet Activating Factor/Platelet Activating Factor Receptor (PAF/PAFr) system plays a role in modulating pain signalling. Our results furthermore indicated the differentiation of the three control groups used (family members, an age-matched group, and young individuals) and the FMS patients (Additional file 1: Figure S3). Statistical assessment of the outcome of a supervised PLS-DA model confirmed the complete separation between the FMS and young control groups. Good model fit values substantiated some unique differences between the global metabolic profiles of the FMS patients and the healthy young controls. The metabolites principally responsible for the differentiation between our FMS patients and controls included taurine and TMAO which were also reported to be significantly increased (*p* < 0.05) in an FMS patient group in a preliminary targeted NMR study [12]. In addition, we observed perturbed succinic acid suggesting altered energy metabolism in FMS. This result is linked to a study [13] where there was relatively elevated: glucose, the glycolytic intermediate phosphoenolpyruvate, pyruvate and nicotinamide adenine dinucleotide (NAD^+^) seen in dried blood spots from FMS patients. This observation was previously reported for patients with chronic widespread pain [35].

A common thread in the metabolomics studies on FMS discussed here is the affirmation of the ability of metabolomics to identify distinct metabolic profiles for FMS patients relative to controls. Some metabolites/biomarkers could therefore contribute to the disease phenotype by having a role in the pathogenesis of FMS. The biomarkers revealed in these metabolomics studies seemed, however, not to be metabolically closely linked, but may be due to the multi-factorial nature of FMS. Noteworthy also are the two main limitations of our own and the other two metabolomics studies: the FMS groups investigated and analytical methods used. Most metabolomics studies are limited by the number of experimental subjects available for investigation, and therefore ultimately call for follow-up validation studies with larger and better-defined experimental groups. Further, given the complexity of the human metabolome and the multi-dimensional nature of biofluids and other biological samples available for metabolomics studies, no single analytical technology can fully disclose and account for the information encapsulated in these samples. Nonetheless, metabolomics retains a promise well beyond the scope of standard clinical chemistry techniques, for affording detailed characterization of metabolic phenotypes and is believed, eventually, to lead to so-called precision medicine in which knowledge of their unique metabolic derangements explains the disease state of individual patients [36]. A third limitation in the present study is the use of the 1990 criteria for FMS (1, 14) as the patient selection was one before publication of the revised criteria in 2011. The use of the revised criteria is now standard practice in our pain clinic.

So, can metabolomics studies contribute to the advancement of objective clinical diagnosis of FMS? The results of the present and the two other metabolomics studies on the disease imply that they can, albeit with qualifications. The analyses of blood spots from FMS patients provided information using IRMS technology that differentiated samples from FMS subjects from those with RA or OA with zero misclassifications (100% accuracy). The accuracy of the metabolomics approach was 75%, but with the advantage of disclosing a prioritized list of metabolites that may underlie the differences identified [9]. The possible role of lysoPCs as biomarkers or as contributors to the FMS phenotype and function in the pathogenesis of this condition suggest they are potential new disease biomarkers and thereby open a new approach for the treatment in FMS [10]. Likewise, the predictive potential of the combination of succinic acid, taurine and creatine proved to be excellent for discriminating between our cases of FMS and controls (AUC = 90%). The combination of creatine and succinic acid also showed a significant correlation with the characteristic symptoms of pain and fatigue in FMS. The inclusion of this predictive information on these three metabolites could in time be considered to form part of the initial evaluation of patients suspected of suffering from the disease, in anticipation of validation of FMS diagnostic markers.

Finally, the involvement of gut microbial–host metabolic perturbations in FMS may prove to contribute significantly in defining the clinical profile in FMS. In health, brain-gut interactions are crucial in the maintaining of homeostasis [37]. It appears that neuroplasticity-related systems and neurotransmitter systems are influenced by the gut–brain axis regulation and perturbed homeostasis may contribute to risk of disease through alterations in gastrointestinal tract, central nervous, autonomic nervous and immune systems [38]. The frequent comorbidity of fibromyalgia with stress related disorders, such as chronic fatigue and irritable bowel syndromes and some CNS related abnormalities, suggests that gut–brain axis regulation may at least be a partial common denominator for these disorders. This view may well be revealed by data from a follow-up targeted metabolomics investigation of high sensitivity, like through mass spectrometric-based technologies.

## Conclusions

An untargeted ^1^H NMR metabolomics analysis of urine samples obtained from a group of clinically well-defined female FMS patients with no psychiatric co-morbidity could be fully differentiated from a group of young healthy women. The presence of metabolic indicators of perturbations in the gut microbiome (hippuric, 2-hydroxyisobutyric and lactic acids) supports the paradigm that regulation of the gut-brain axis becomes affected in stress related disorders, like FMS. Three metabolite markers (taurine, creatine and succinic acid) were important for the differentiation between FMS patients and controls and were significant indicators of the pain and fatigue symptoms in FMS. ROC analysis and odds ratios substantiated the good predictive potential of a combination of these three metabolites for FMS in the present patient group. Follow-up metabolomics research on a larger number of urine samples, including those from individuals at high risk of developing the disease, as well as longitudinal studies on FMS patients during treatment, are needed to validate the findings presented here and to potentially detect effects which would require greater statistical power. These markers may in time provide objective supplementary information together with tender-point measurements and FIQR questionnaires used to confirm FMS.

## Additional files

Additional file 1: Supplementary information (SI) (PDF providing detailed descriptions of methods and additional information – clinical, spectral and statistical – to support the manuscript). (DOCX 1270 kb)

Additional file 2: Raw data matrix (Raw NMR spectral data (Excel format) normalized relative to the CH_3_ singlet of creatinine at 3.13 ppm). (XLSX 298 kb)

## Abbreviations

^1^H–NMR: Proton nuclear magnetic resonance
ACR: American College of Rheumatology
AUC: Area under the ROC curve
AUROC: Area under the ROC curve
CF: Group of first-degree relatives of the patients
CN: Group of healthy young subjects
CNS: Central nervous system
CO: Group of age-matched subjects but unrelated to the patients
CV AUROC: Cross-validated AUROC
CWP: Chronic widespread pain
ES: Effect size
FC: Fold change
FIQR: Fibromyalgia Impact Questionnaire
FM: Fibromyalgia
FMS: Fibromyalgia syndrome
IHCQ: In-house clinical questionnaire
IRMS: Mid-infrared micro-spectroscopy
LC/Q-TOF/MS: liquid chromatography/quadrupole–time of flight/mass spectrometry
LOO CV: Leave-one-out cross-validation
LV: Latent variable
lysoPCs: Lysophosphocholines
MW: Mann–Whitney test
N-acetyl-X: N-acetyl-derivative
NTeMBI: Nuclear Technologies in Medicine and Biosciences Initiative
NWU: North-West University
OA: Osteoarthritis
OR: Odds ratio
PAF/PAFr: Platelet Activating Factor/Platelet Activating Factor Receptor
PC: Principal component
PCA: Principal components analysis
PLS-DA: Partial least squares discriminant analysis
Q^2^: Predictive ability parameter
R^2^: Goodness-of-fit parameter
RA: Rheumatoid arthritis
ROC: Receiver Operating Characteristic
SN: Substantia nigra
SUM-3: Numerical sum of the concentrations of taurine, creatine and TMAO
TMAO: Trimethylamine-N-oxide
TPs: Tender-points
VIP: Variable important in projection

## References

[1] Wolfe F, Smythe HA, Yunus MB, Bennett RM, Bombardier C, Goldenberg DL (1990). The American College of Rheumatology 1990 criteria for the classification of fibromyalgia. Arthritis Rheum.

[2] Wolfe F, Clauw DJ, Fitzcharles MA, Goldenberg DL, Katz RS, Mease P (2010). The American College of Rheumatology preliminary diagnostic criteria for fibromyalgia and measurement of symptom severity. Arthritis Care Res.

[3] Branco JC, Bannwarth B, Failde I, Carbonell JA, Blotman F, Spaeth M, Saraiva F (2010). Prevalence of fibromyalgia: a survey in five European countries. Semin Arthritis Rheum.

[4] Lawrence RC, Felson DT, Helmick CG, Arnold LM, Choi H, Deyo RA (2008). Estimates of the prevalence of arthritis and other rheumatic conditions in the United States: part II. Arthritis Rheum.

[5] Russell IJ, Orr MD, Littman B, Vipraio GA, Alboukrek D, Michalek JE (1994). Elevated cerebrospinal fluid levels of substance P in patients with the fibromyalgia syndrome. Arthritis Rheum.

[6] Yunus MB, Dailey JW, Aldag JC, Masi AT, Jobe PC (1992). Plasma and urinary catecholamines in primary fibromyalgia: a controlled study. J Rheumatol.

[7] Legangneux E, Mora JJ, Spreux-Varoquaux O, Thorin I, Herrou M, Alvado G (2001). Cerebrospinal fluid biogenic amine metabolites, plasma-rich platelet serotonin and [3H]imipramine reuptake in the primary fibromyalgia syndrome. Rheumatol.

[8] Meyer HP (2002). Myofascial pain syndrome and its suggested role in the pathogenesis and treatment of fibromyalgia syndrome. Curr Pain Headache Rep.

[9] Levine JS, Burakoff R (2011). Extraintestinal manifestations of inflammatory bowel disease. Gastroenterol Hepatol (NY).

[10] Clauw DJ (2015). Fibromyalgia and related conditions. Mayo Clin Proc Elsevier.

[11] Dumas M-E, Davidovic L (2013). Metabolic phenotyping and systems biology approaches to understanding neurological disorders. F1000Prime Rep.

[12] Richards SCM, Bell J, Cheung Y-L, Cleare A, Scott DL (2001). Abstract 382: muscle metabolites detected in urine in fibromyalgia and chronic fatigue syndrome may suggest ongoing muscle damage.

[13] Hackshaw KV, Rodriguez-Saona L, Plans M, Bell LN, Buffington CAT (2013). A bloodspot-based diagnostic test for fibromyalgia syndrome and related disorders. Analyst.

[14] Caboni P, Liori B, Kumar A, Santoru ML, Asthana S, Pieroni E (2014). Metabolomics analysis and modeling suggest a Lysophosphocholines-PAF receptor interaction in fibromyalgia. PLoS One.

[15] Giacomelli C, Sernissi F, Rossi A, Bombardieri S, Bazzichi L (2014). Biomarkers in fibromyalgia: a review. Curr Biomark Find.

[16] Xiao Y, Russell IJ, Liu YG (2012). A brain-derived neurotrophic factor polymorphism Val66Met identifies fibromyalgia syndrome subgroup with higher body mass index and C-reactive protein. Rheumatol Int.

[17] Bennett RM, Friend R, Jones KD, Ward R, Han BK, Ross RL (2009). The revised fibromyalgia impact questionnaire (FIQR): validation and psychometric properties. Arthritis Res Ther.

[18] Engelke UF, Liebrand-van Sambeek ML, De Jong JG, Leroy JG, Morava É, Smeitink JA (2004). N-acetylated metabolites in urine: proton nuclear magnetic resonance spectroscopic study on patients with inborn errors of metabolism. Clin Chem.

[19] Wevers RA, Engelke U, Rotteveel JJ, Heerschap A, De Jong JG, Abeling NG (1997). ^1^H NMR spectroscopy of body fluids in patients with inborn errors of purine and pyrimidine metabolism. J Inherit Metab Dis.

[20] Viant MR, Lyeth BG, Miller MG, Berman RF (2005). An NMR metabolomic investigation of early metabolic disturbances following traumatic brain injury in a mammalian model. NMR Biomed.

[21] Worley B, Powers R (2013). Multivariate analysis in metabolomics. Curr Metabolomics.

[22] Ditre CM, Griffin TD, Murphy GF, Sueki H, Telegan B, Johnson WC (1996). Effects of 2-hydroxy acids on photoaged skin: a pilot clinical, histologic, and ultrastructural study. J Am Acad Dermatol.

[23] Pellerin L, Pellegri G, Bittar PG, Charnay Y, Bouras C, Martin JL (1998). Evidence supporting the existence of an activity-dependent astrocyte-neuron lactate shuttle. Dev Neurosci.

[24] Huxtable RJ (1992). Physiological actions of taurine. Physiol Rev.

[25] Morales I, Dopico JG, Sabate M, Gonzalez-Hernandez T, Rodriguez M (2007). Substantia nigra osmoregulation: taurine and ATP involvement. Am J Physiol Cell Physiol.

[26] Solanky KS, Bailey NJ, Beckwith-Hall BM, Bingham S, Davis A (2005). Biofluid 1H NMR-based metabonomic techniques in nutrition research - metabolic effects of dietary isoflavones in humans. J Nutr Biochem.

[27] Bertini I, Calabro A, De Carli V, Luchinat C, Nepi S, Porfirio B, Tenori L (2008). The metabonomic signature of celiac disease. J Proteome Res.

[28] Li M, Wang B, Zhang M, Rantalainen M, Wang S, Zhou H (2008). Symbiotc gut microbes modulate human metabolic phenotypes. Proc Natl Acad Sci U S A.

[29] Mason S, Reinecke CJ, Kulik W, van Cruchten A, Solomons R, van Furth MT (2016). Cerebrospinal fluid in tuberculous meningitis exhibits only the L-enantiomer of lactic acid. BMC Infect Dis.

[30] Field AP (2009). Discovering statistics using IBM SPSS statistics: and sex and drugs and rock 'n' roll.

[31] Thompson B (2001). Significance, effect sizes, stepwise methods, and other issues: strong arguments move the field. J Exp Educ.

[32] Cohen J (1988). Statistical power analysis of the behavioural sciences.

[33] Quinones MP, Kaddurah-Daouk R (2009). Metabolomics tools for identifying biomarkers for neuropsychiatric diseases. Neurobiol Dis.

[34] Sinclair AJ, Viant MR, Ball AK, Burdon MA, Walker EA, Stewart PM (2010). NMR-based metabolomic analysis of cerebrospinal fluid and serum in neurological diseases- a diagnostic tool?. NMR Biomed.

[35] Mäntyselkä P, Miettola J, Niskanen L, Kumpusalo E (2008). Glucose regulation and chronic pain at multiple sites. Rheumatology.

[36] Clish CB (2015). Metabolomics: an emerging but powerful tool for precision medicine. Mol Case Stud.

[37] Mayer EA, Tillisch K. The brain-gut axis in abdominal pain syndromes. Ann Rev Med. 2011;62 10.1146/annurev-med-012309-103958 PMC3817711 21090962

[38] Cryan JF, O’Mahony SM (2011). The microbiome-gut-brain axis: from bowel to behavior. Neurogastroenterol Motil.

