# Supplementary material for: A diagnostic biomarker profile for fibromyalgia syndrome based on an NMR metabolomics study of selected patients and controls
Source: BMC Neurol. 2017 May 11;17:88. doi: 10.1186/s12883-017-0863-9 (PMC5426044; doi:10.1186/s12883-017-0863-9)
Supplement: Supplementary file 1 — Supplementary information (SI) (PDF providing detailed descriptions of methods and additional information – clinical, spectral and statistical – to support the manuscript). (DOCX 1270 kb) [file 12883_2017_863_MOESM1_ESM.docx]

**Supplementary Material**

**A diagnostic biomarker profile for Fibromyalgia Syndrome based on an NMR metabolomics study of selected patients and controls**

Bontle G. Malatji^1^, Helgard Meyer^2^, Shayne Mason^1^, Udo F.H. Engelke^3^, Ron A. Wevers^3^, Mari van Reenen^1^ and Carolus J. Reinecke^1^

^1^Centre for Human Metabonomics, Faculty of Natural Sciences, North-West University (Potchefstroom Campus), Private Bag X6001, Potchefstroom, South Africa,

^2^Department of Family Medicine, Kalafong Hospital, University of Pretoria, Private Bag X396, Pretoria, South Africa

^3^Radboud University Nijmegen Medical Centre, Translational Metabolic Laboratory, Department of Laboratory Medicine, PO Box 9101, 6500 HB Nijmegen, The Netherlands,

Contents

[S1: Experimental subjects symptom assessment questionnaire 2](#_Toc450591339)

[A - Fibromyalgia Impact Questionnaire (FIQR) 2](#_Toc450591340)

[B - Clinical Questionnaire 3](#_Toc450591341)

[S2: Comparison of FMS symptom severity with a published reference group 8](#_Toc450591342)

[S3: Correlation table based on the FIQR questionnaire 10](#_Toc450591343)

[S4: Normalized data of the original NMR spectral bins 16](#_Toc450591344)

[S5: Outlier identification 17](#_Toc450591345)

[S6: PCA Analysis 18](#_Toc450591346)

[S7: *N*-acetyl aspartic acid (NAA) verification 19](#_Toc450591347)

[Overview 19](#_Toc450591348)

[Reagents 19](#_Toc450591349)

[Sample preparation and analysis 19](#_Toc450591350)

[Results and discussion 19](#_Toc450591351)

[References: 21](#_Toc450591352)

# S1: Experimental subjects symptom assessment questionnaire

The Fibromyalgia Impact Questionnaire (FIQR) is an internationally derived questionnaire developed by Burckhardt and co-workers [1]. It was developed with the aim to evaluate and understand the effects of therapy on the broad range of symptoms that manifest in FMS. As such, the questionnaire has routinely been used, since its official release in 1991, as a means to assess the progression of the disorder and any therapeutic interventions applied [2].

Questionnaire A in Table S1 shows this FIQR questionnaire that was voluntarily completed by the FMS patients who took part in this study. The in-house clinical questionnaire, Table S1B, was drawn up to identify secondary data about the patients for use in conjunction with the FIQR questionnaire.

Table S1: Fibromyalgia Impact Questionnaire (FIQR) (A) and Clinical questionnaire (B). Questionnaire A was used by the clinicians to assess the severity of the symptoms experienced by the FMS patients. Questionnaire B was used to gather supplementary information on the FMS patients

## A - Fibromyalgia Impact Questionnaire (FIQR)

1. ***Function domain***

**Directions:** For each question, place an “X” in the box that best indicates how much your fibromyalgia made it difficult to do each of the following activities during the past 7 days

| Brush or comb your hair | ***No difficulty*** | **🗌0 🗌1 🗌2 🗌3 🗌4 🗌5 🗌6 🗌7 🗌8 🗌9 🗌10** | ***Very difficult*** |
| --- | --- | --- | --- |
|  |  | | |
| Walk continuously for 20 minutes | ***No difficulty*** | **🗌0 🗌1 🗌2 🗌3 🗌4 🗌5 🗌6 🗌7 🗌8 🗌9 🗌10** | ***Very difficult*** |
|  |  | | |
| Prepare a homemade meal | ***No difficulty*** | **🗌0 🗌1 🗌2 🗌3 🗌4 🗌5 🗌6 🗌7 🗌8 🗌9 🗌10** | ***Very difficult*** |
|  |  | | |
| Vacuum, scrub or sweep floors | ***No difficulty*** | **🗌0 🗌1 🗌2 🗌3 🗌4 🗌5 🗌6 🗌7 🗌8 🗌9 🗌10** | ***Very difficult*** |
|  |  | | |
| Lift and carry a bag full of groceries | ***No difficulty*** | **🗌0 🗌1 🗌2 🗌3 🗌4 🗌5 🗌6 🗌7 🗌8 🗌9 🗌10** | ***Very difficult*** |
|  |  | | |
| Climb one flight of stairs | ***No difficulty*** | **🗌0 🗌1 🗌2 🗌3 🗌4 🗌5 🗌6 🗌7 🗌8 🗌9 🗌10** | ***Very difficult*** |
|  |  | | |
| Change bed sheets | ***No difficulty*** | **🗌0 🗌1 🗌2 🗌3 🗌4 🗌5 🗌6 🗌7 🗌8 🗌9 🗌10** | ***Very difficult*** |
|  |  | | |
| Sit in a chair for 45 minutes | ***No difficulty*** | **🗌0 🗌1 🗌2 🗌3 🗌4 🗌5 🗌6 🗌7 🗌8 🗌9 🗌10** | ***Very difficult*** |
|  |  | | |
| Go shopping for groceries | ***No difficulty*** | **🗌0 🗌1 🗌2 🗌3 🗌4 🗌5 🗌6 🗌7 🗌8 🗌9 🗌10** | ***Very difficult*** |
|  |  |  |  |

1. ***Overall impact domain:***

**Directions:** For each question, check the one box that best describes the overall impact of your fibromyalgia over the last 7 days:

| Fibromyalgia prevented me from accomplishing goals for the week | ***Never*** | **🗌0 🗌1 🗌2 🗌3 🗌4 🗌5 🗌6 🗌7 🗌8 🗌9 🗌10** | ***Always*** |
| --- | --- | --- | --- |
|  |  | | |
| I was completely overwhelmed by my fibromyalgia symptoms | ***Never*** | **🗌0 🗌1 🗌2 🗌3 🗌4 🗌5 🗌6 🗌7 🗌8 🗌9 🗌10** | ***Always*** |
|  |  |  |  |

1. ***Symptoms domain:***

**Directions:** For each of the following 10 questions, select the one circle that best indicates the intensity of your fibromyalgia symptoms over the past 7 days

| Please rate your level of pain | ***No pain*** | **🗌0 🗌1 🗌2 🗌3 🗌4 🗌5 🗌6 🗌7 🗌8 🗌9 🗌10** | ***Unbearable pain*** |
| --- | --- | --- | --- |
| Please rate your level of energy | ***Lots of energy*** | **🗌0 🗌1 🗌2 🗌3 🗌4 🗌5 🗌6 🗌7 🗌8 🗌9 🗌10** | ***No energy*** |
| Please rate your level of stiffness | ***No stiffness*** | **🗌0 🗌1 🗌2 🗌3 🗌4 🗌5 🗌6 🗌7 🗌8 🗌9 🗌10** | ***Severe stiffness*** |
| Please rate the quality of your sleep | ***Awoke well rested*** | **🗌0 🗌1 🗌2 🗌3 🗌4 🗌5 🗌6 🗌7 🗌8 🗌9 🗌10** | ***Awoke very tired*** |
| Please rate your level of depression | ***No depression*** | **🗌0 🗌1 🗌2 🗌3 🗌4 🗌5 🗌6 🗌7 🗌8 🗌9 🗌10** | ***Very depressed*** |
| Please rate your level of memory problems | ***Good memory*** | **🗌0 🗌1 🗌2 🗌3 🗌4 🗌5 🗌6 🗌7 🗌8 🗌9 🗌10** | ***Very poor memory*** |
| Please rate your level of anxiety | ***Not anxious*** | **🗌0 🗌1 🗌2 🗌3 🗌4 🗌5 🗌6 🗌7 🗌8 🗌9 🗌10** | ***Very anxious*** |
|  | | | |
| Please rate your level of tenderness to touch | ***No tenderness*** | **🗌0 🗌1 🗌2 🗌3 🗌4 🗌5 🗌6 🗌7 🗌8 🗌9 🗌10** | ***Very tender*** |
| Please rate your level of balance problems | ***No imbalance*** | **🗌0 🗌1 🗌2 🗌3 🗌4 🗌5 🗌6 🗌7 🗌8 🗌9 🗌10** | ***Severe imbalance*** |
| Please rate your level of sensitivity to loud noises, bright lights, odors and cold | ***No sensitivity*** | **🗌0 🗌1 🗌2 🗌3 🗌4 🗌5 🗌6 🗌7 🗌8 🗌9 🗌10** | ***Extreme sensitivity*** |
|  |  |  |  |
|  |  |  |  |

## B - Clinical Questionnaire

| 1. Age: | |  | | | Years | | |  | | Months | | |
| --- | --- | --- | --- | --- | --- | --- | --- | --- | --- | --- | --- | --- |
|  | | | | | | | | | | | | |
| 1. Relationship status: | | 1. Married | | 2. Engaged | | | 3. In a relationship, but not married or engaged | | | | 4. Single | |
|  | | | | | | | | | | | | |
| 1. Current employment status | 1. Fulltime employed | | 2. Part-time employed | | | 3. Home executive (housewife) | | | 4. Retired | | | 5. Disabled |

|  | | | | | | | | |
| --- | --- | --- | --- | --- | --- | --- | --- | --- |
| 1. How long ago did your fibromyalgia symptoms start (e.g. widespread muscle pain, poor sleep, fatigue, headaches, etc.) | |  | | | Years |  | Months | |
|  | | | | | | | | |
| 1. How long ago were you diagnosed with fibromyalgia? | |  | | | Years |  | Months | |
|  | | | | | | | | |
| 1. (a) Were your fibromyalgia symptoms triggered by? Please tick (√)   (you may tick more than one block) | | | 1. Neck injury | | | | |  |
|  |  |  | 2. Other injuries | | | | |  |
|  |  |  | 3. After surgical procedure | | | | |  |
|  |  |  | 4. Severe emotional stress | | | | |  |
|  |  |  | 5. Acute infection | | | | |  |
|  |  |  | 6. Spontaneous onset | | | | |  |
|  |  |  | 7. Uncertain | | | | |  |
|  | | | | | | | | |
| (b) | Length | | |  | | | | |
|  | Bodyweight | | |  | | | | |

1. Please rate your pain by circling the **one** number that best describes you pain at its **worst** in the last month. (A rating of 10 would indicate pain so severe as to prohibit all activity; the worst pain you can imagine.)


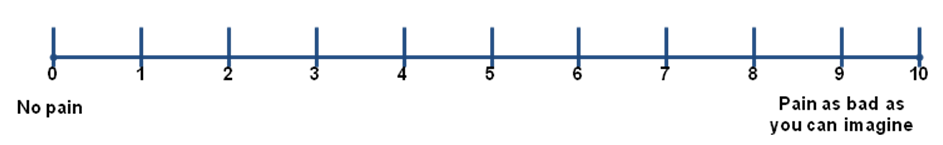


1. Please rate your pain by circling the **one** number that best describes you pain on the **average** in the last month. (A rating of 10 would indicate pain so severe as to prohibit all activity; the worst pain you can imagine.)


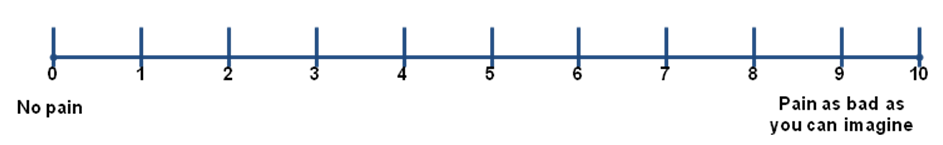


1. (i) Which medications are you receiving **for your pain**?

Please tick

| Trepeline |  | Lyrica |  |
| --- | --- | --- | --- |
| Cymbalta |  | Syndol |  |
| Tramal (Tramahexal) |  | Myprodol |  |
| Tenston |  | Mypaid |  |
| Stilpayne |  | Cataflam |  |
| Other |  | | |

(ii) How often do you take pain killers (e.g. Tramal, Tramahexal, Tramacet, Panado, Syndol., Mypaid, Cataflam, Voltaren, etc.)

| ± Once a week |  |
| --- | --- |
| ± Twice a week |  |
| ± Three days a week |  |
| ± Every second day |  |
| ± Daily |  |

| 10. | During the past week how much did the state of your health, including any pain, interfere with the following things: choose the one number, from 0 to 4 below, that best describes your state and write them in the appropriate box (I to vi).   1. Not at all 2. A little bit 3. Moderately 4. Quite a bit 5. Extremely | |
| --- | --- | --- |
| 1. Mood | |  |
| 1. Relations with other people | |  |
| 1. Walking ability | |  |
| 1. Sleep | |  |
| 1. Normal Work (includes both work outside the home and housework) | |  |
| 1. Enjoyment of life | |  |

|  | | | | | |
| --- | --- | --- | --- | --- | --- |
| 1. Have you ever been diagnosed and treated for **depression** |  | Yes | |  | No |
|  | | | | | |
| 1. Do you suffer from regular **headaches?** |  | | Yes |  | No |
|  | | | | | |
| 1. **Irritable bowel syndrome** (IBS) is known to commonly affect patients with fibromyalgia. It is characterized by abdominal pain and cramps as well as bloating, flatulence, diarrhea and/or constipation | | | | | |
|  | | | | | |
| Have you ever been diagnosed with IBS? |  | | Yes |  | No |

|  | | | | | | | | | |
| --- | --- | --- | --- | --- | --- | --- | --- | --- | --- |
| 1. **Restless leg syndrome** (RLS) is characterized by uncomfortable sensations in the lower legs and an uncontrollable urge to move them so as to provide relief. Some of the sensations felt in RLS include burning, creeping or a crawling feeling inside the legs. | | | | | | | | | |
|  | | | | | | | | | |
| Have you ever experienced symptoms of RLS? | | | |  | Yes | |  | | No |
|  | | | | | | | | | |
| 15. Have you suffered from **anxiety** since being diagnosed with fibromyalgia or thereafter? (Symptoms such as feeling nervous most of the time, not able to control worrying, etc.) | |  | Yes | | |  | | | No |
|  | | | | | | | | | |
| 16. If yes, have you been diagnosed and treated for anxiety? | |  | Yes | | |  | | | No |
|  | | | | | | | | | |
| 1. Have you ever suffered from a **sleep disturbance**? | |  | Yes | | |  | | | No |
|  | | | | | | | | | |
| If yes, please tick the appropriate block(s)  (you may tick more than one block) | Problem with sleep initiation | | | | | | |  | |
|  | Problem with maintaining sleep | | | | | | |  | |
|  | Early morning awakening | | | | | | |  | |
|  | Waking up feeling unrefreshed | | | | | | |  | |
|  | | | | | | | | | |
| 1. **Dysmenorrhoea** is defined as painful menstruation often associated with cramps for mostly 1–3 days after beginning of menstruation. | | | | | | | | | |
| Were you treated before or are you currently being treated for dysmenorrhoea? | | | | | Yes | | |  | |
|  | | | | | No | | |  | |

Table S2: Summary of the supplementary data collected based on the in-house questionnaire (Table S1B) completed by the FMS patient group.

| **Variable** | **Mean**  **(SD)** | **%** |
| --- | --- | --- |
| **1. Socio-demographic information** | | |
| Age (years) | 45.5 | n/a |
| Marital status  Single  Separated/Divorced/Widow  In permanent relationship  Married | 2  0  2  15 | 11  0  11  78 |
| Employment  Disabled/Retired  Housewife  Part-time  Full-time | 2  6  1  10 | 11  32  5  52 |
| **2. Pain experience** | | |
| Worst pain experience | 7,7 | n/a |
| Recent past pain average | 5,7 | n/a |
| Pain specific medication  Trepiline (antidepressant for neuropathic pain)  Cymbalta (antidepressant for chronic pain)  Tramal (analgesic for moderate to severe pain)  Myprodol (relief of pain of inflammatory origin)  Patients using also other medication against pain | 12  10  9  6  11 | 63  52  47  32  58 |
| **3. Levels of emotional experiences affected by FMS**  Mood  Relations with other people  Enjoyment of life  Normal work | 2.3  2.2  2.1  2.4 | n/a |

**0** = Not at all **1** = A little bit **2** = Moderately  **3** = Quite a bit **4** = Extremely

# S2: Comparison of FMS symptom severity with a published reference group

The mean scores of FMS patients’ symptoms based on severity in relation to the published reference group [3] are shown in Table S3.


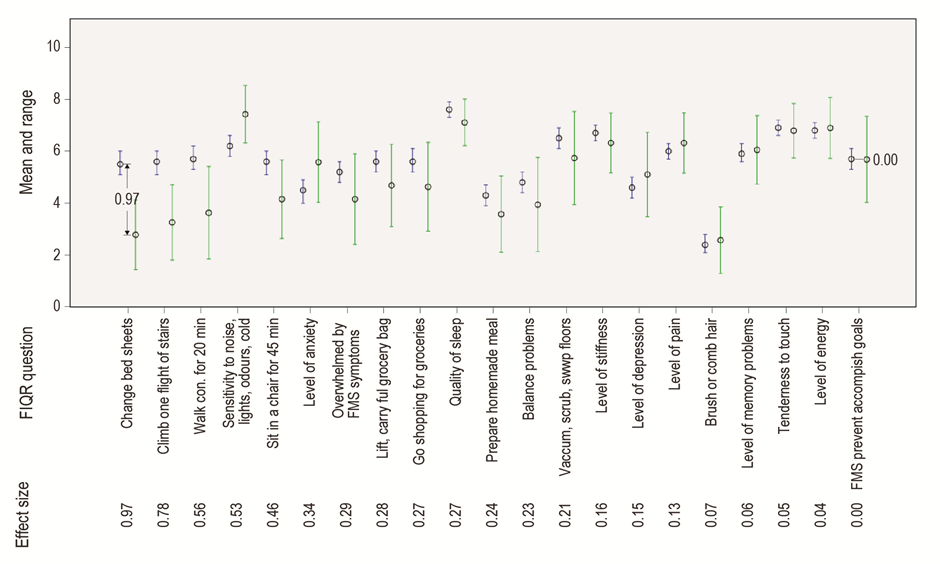


**Figure S1**: Comparison of minimum, mean and maximum scores of fibromyalgia patients’ symptoms based on severity in relation to a published reference group. Every FIQR question is compared with the data from a reference group, presented for the revised FIQR. The questions are ranked according to the difference between the effect sizes (ES) ranked from high to low (1.0 ≥ ES ≥ 0.00), with the mean values shown as a circular point in the figure. The range of points scored for the reference group is shown in blue and for the present FMS group by green vertical bars.

Higher scores are indicative of greater dysfunction or symptom severity, while differences between the blue (previously published study) and green (current study) observed ranges show how the current patient group differs from another patient group. These differences were also quantified using Cohen’s d-value as a measure of practical significance of differences (i.e. effect size). Effect sizes exceeding 0.5 are considered practically visible, whereas those exceeding 0.8 are considered practically significant [4]. The six highest scores reported for the reference group were: sleep quality (mean 7.61 ± 2.4 (standard deviation)), tenderness to touch (6.86 ± 2.5), energy level (6.80 ± 2.4), stiffness (6.72 ± 2.2), sensitivity to the environment (6.19 ± 2.9), and pain (6.01 ± 2.1). The mean values of the highest scores obtained for the present FMS group remarkably resemble the published observations: sensitivity to the environment > sleep quality > energy level > tenderness to touch > stiffness and pain. Likewise, ‘difficulty with combing hair' had the lowest score in both groups. The scores for the three FIQR domains did, however, indicate some clear differences between the two groups: Reliability was assessed using Cronbach’s alpha coefficient (α) and indicated reliability for all domains. The mean values (reference group vs present group) obtained were 18 vs 36 for the ‘Function domain’ (α = 0.94 and mean inter-item correlation = 0.62); 11 vs 10 for the ‘Impact domain’ (α = 0.89 and mean inter-item correlation = 0.8) and 30 vs 60 for the ‘Symptoms domain’ (α = 0.88 and mean inter-item correlation = 0.44). The total scores (α = 0.95 and mean inter-item correlation = 0.5) were comparable: 55 vs 50. It is important, finally, to notice that the difference between the minimum and maximum scores obtained for all 21 questions involving the present group mostly exceeded the values for the reference group, clearly suggesting a greater diversity between the present FMS patients group than in the reference group.

# S3: Correlation table based on the FIQR questionnaire

**Table S3**: Correlation table data, as based on the FIQR questionnaire, used to draw up Fig. 1 in the main article.

| **Comparison** | **Correlation coefficient** | **Correlation coefficient** | ***p*-value** |
| --- | --- | --- | --- |
|  |  | **(As quoted in Fig. 1**  **of main article)** |  |
| Hair correlation with Walk | 0.466454 | 0.5 | 0.012 |
| Hair correlation with Meals | 0.418437 | 0.4 | 0.024 |
| Hair correlation with Clean | 0.460269 | 0.5 | 0.014 |
| Hair correlation with Carry | 0.421214 | 0.4 | 0.022 |
| Hair correlation with Stairs | 0.216187 | 0.2 | 0.247 |
| Hair correlation with Make bed | 0.177345 | 0.2 | 0.345 |
| Hair correlation with Sit | 0.307686 | 0.3 | 0.095 |
| Hair correlation with Shop | 0.269565 | 0.3 | 0.154 |
| Hair correlation with Goals | 0.335035 | 0.3 | 0.070 |
| Hair correlation with Symptoms | 0.195924 | 0.2 | 0.296 |
| Hair correlation with Pain | 0.261516 | 0.3 | 0.159 |
| Hair correlation with Energy | 0.068820 | 0.1 | 0.710 |
| Hair correlation with Stiffness | 0.497131 | 0.5 | 0.007 |
| Hair correlation with Sleep | 0.157715 | 0.2 | 0.407 |
| Hair correlation with Depression | 0.014139 | 0.0 | 0.940 |
| Hair correlation with Memory | 0.048174 | 0.0 | 0.795 |
| Hair correlation with Anxiety | -0.151901 | -0.2 | 0.414 |
| Hair correlation with Tenderness | 0.202921 | 0.2 | 0.279 |
| Hair correlation with Balance | 0.079710 | 0.1 | 0.676 |
| Hair correlation with Environment | 0.020921 | 0.0 | 0.911 |
| Walk correlation with Meals | 0.538961 | 0.5 | 0.003 |
| Walk correlation with Clean | 0.719570 | 0.7 | 0.000 |
| Walk correlation with Carry | 0.636686 | 0.6 | 0.000 |
| Walk correlation with Stairs | 0.646953 | 0.6 | 0.000 |
| Walk correlation with Make bed | 0.483494 | 0.5 | 0.008 |
| Walk correlation with Sit | 0.614890 | 0.6 | 0.001 |
| Walk correlation with Shop | 0.557361 | 0.6 | 0.002 |
| Walk correlation with Goals | 0.550165 | 0.6 | 0.002 |
| Walk correlation with Symptoms | 0.609392 | 0.6 | 0.001 |
| Walk correlation with Pain | 0.553749 | 0.6 | 0.002 |
| Walk correlation with Energy | 0.403911 | 0.4 | 0.025 |
| Walk correlation with Stiffness | 0.620928 | 0.6 | 0.001 |
| Walk correlation with Sleep | 0.149298 | 0.1 | 0.420 |
| Walk correlation with Depression | 0.281064 | 0.3 | 0.124 |
| Walk correlation with Memory | 0.312705 | 0.3 | 0.083 |
| Walk correlation with Anxiety | 0.078433 | 0.1 | 0.665 |
| Walk correlation with Tenderness | 0.549777 | 0.5 | 0.003 |
| Walk correlation with Balance | 0.342981 | 0.3 | 0.065 |
| Walk correlation with Environment | 0.389492 | 0.4 | 0.033 |
| Meals correlation with Clean | 0.666757 | 0.7 | 0.000 |
| Meals correlation with Carry | 0.546649 | 0.5 | 0.002 |
| Meals correlation with Stairs | 0.448906 | 0.4 | 0.014 |
| Meals correlation with Make bed | 0.718525 | 0.7 | 0.000 |
| Meals correlation with Sit | 0.569582 | 0.6 | 0.002 |
| Meals correlation with Shop | 0.443203 | 0.4 | 0.016 |
| Meals correlation with Goals | 0.563110 | 0.6 | 0.002 |
| Meals correlation with Symptoms | 0.563025 | 0.6 | 0.002 |
| Meals correlation with Pain | 0.508146 | 0.5 | 0.005 |
| Meals correlation with Energy | 0.469058 | 0.5 | 0.009 |
| Meals correlation with Stiffness | 0.411774 | 0.4 | 0.023 |
| Meals correlation with Sleep | 0.278236 | 0.3 | 0.133 |
| Meals correlation with Depression | 0.267680 | 0.3 | 0.143 |
| Meals correlation with Memory | 0.175897 | 0.2 | 0.331 |
| Meals correlation with Anxiety | 0.176474 | 0.2 | 0.330 |
| Meals correlation with Tenderness | 0.245081 | 0.2 | 0.180 |
| Meals correlation with Balance | 0.377279 | 0.4 | 0.042 |
| Meals correlation with Environment | 0.237656 | 0.2 | 0.193 |
| Clean correlation with Carry | 0.778044 | 0.8 | 0.000 |
| Clean correlation with Stairs | 0.416107 | 0.4 | 0.024 |
| Clean correlation with Make bed | 0.505193 | 0.5 | 0.006 |
| Clean correlation with Sit | 0.723825 | 0.7 | 0.000 |
| Clean correlation with Shop | 0.716828 | 0.7 | 0.000 |
| Clean correlation with Goals | 0.684344 | 0.7 | 0.000 |
| Clean correlation with Symptoms | 0.653202 | 0.7 | 0.000 |
| Clean correlation with Pain | 0.695425 | 0.7 | 0.000 |
| Clean correlation with Energy | 0.456994 | 0.5 | 0.012 |
| Clean correlation with Stiffness | 0.578102 | 0.6 | 0.002 |
| Clean correlation with Sleep | 0.241471 | 0.2 | 0.198 |
| Clean correlation with Depression | 0.326561 | 0.3 | 0.078 |
| Clean correlation with Memory | 0.377517 | 0.4 | 0.039 |
| Clean correlation with Anxiety | 0.186056 | 0.2 | 0.310 |
| Clean correlation with Tenderness | 0.316500 | 0.3 | 0.087 |
| Clean correlation with Balance | 0.474216 | 0.5 | 0.012 |
| Clean correlation with Environment | 0.248322 | 0.2 | 0.178 |
| Carry correlation with Stairs | 0.424982 | 0.4 | 0.019 |
| Carry correlation with Make bed | 0.399043 | 0.4 | 0.029 |
| Carry correlation with Sit | 0.576935 | 0.6 | 0.001 |
| Carry correlation with Shop | 0.645120 | 0.6 | 0.000 |
| Carry correlation with Goals | 0.685912 | 0.7 | 0.000 |
| Carry correlation with Symptoms | 0.656023 | 0.7 | 0.000 |
| Carry correlation with Pain | 0.703284 | 0.7 | 0.000 |
| Carry correlation with Energy | 0.541981 | 0.5 | 0.003 |
| Carry correlation with Stiffness | 0.543761 | 0.5 | 0.003 |
| Carry correlation with Sleep | 0.396545 | 0.4 | 0.031 |
| Carry correlation with Depression | 0.192205 | 0.2 | 0.290 |
| Carry correlation with Memory | 0.322608 | 0.3 | 0.073 |
| Carry correlation with Anxiety | 0.110047 | 0.1 | 0.541 |
| Carry correlation with Tenderness | 0.380494 | 0.4 | 0.036 |
| Carry correlation with Balance | 0.353276 | 0.4 | 0.056 |
| Carry correlation with Environment | 0.241913 | 0.2 | 0.182 |
| Stairs correlation with Make bed | 0.539328 | 0.5 | 0.004 |
| Stairs correlation with Sit | 0.473776 | 0.5 | 0.009 |
| Stairs correlation with Shop | 0.512020 | 0.5 | 0.006 |
| Stairs correlation with Goals | 0.421135 | 0.4 | 0.020 |
| Stairs correlation with Symptoms | 0.478117 | 0.5 | 0.010 |
| Stairs correlation with Pain | 0.344401 | 0.3 | 0.059 |
| Stairs correlation with Energy | 0.357647 | 0.4 | 0.050 |
| Stairs correlation with Stiffness | 0.598036 | 0.6 | 0.001 |
| Stairs correlation with Sleep | 0.075891 | 0.1 | 0.685 |
| Stairs correlation with Depression | 0.197297 | 0.2 | 0.285 |
| Stairs correlation with Memory | 0.245055 | 0.2 | 0.179 |
| Stairs correlation with Anxiety | 0.079738 | 0.1 | 0.663 |
| Stairs correlation with Tenderness | 0.397309 | 0.4 | 0.031 |
| Stairs correlation with Balance | 0.488164 | 0.5 | 0.009 |
| Stairs correlation with Environment | 0.382550 | 0.4 | 0.038 |
| Make bed correlation with Sit | 0.522093 | 0.5 | 0.004 |
| Make bed correlation with Shop | 0.437500 | 0.4 | 0.020 |
| Make bed correlation with Goals | 0.428384 | 0.4 | 0.019 |
| Make bed correlation with Symptoms | 0.561697 | 0.6 | 0.003 |
| Make bed correlation with Pain | 0.464860 | 0.5 | 0.011 |
| Make bed correlation with Energy | 0.410963 | 0.4 | 0.025 |
| Make bed correlation with Stiffness | 0.310924 | 0.3 | 0.092 |
| Make bed correlation with Sleep | 0.189484 | 0.2 | 0.315 |
| Make bed correlation with Depression | 0.304500 | 0.3 | 0.101 |
| Make bed correlation with Memory | 0.276221 | 0.3 | 0.133 |
| Make bed correlation with Anxiety | 0.202777 | 0.2 | 0.271 |
| Make bed correlation with Tenderness | 0.363048 | 0.4 | 0.051 |
| Make bed correlation with Balance | 0.581692 | 0.6 | 0.002 |
| Make bed correlation with Environment | 0.423270 | 0.4 | 0.022 |
| Sit correlation with Shop | 0.716204 | 0.7 | 0.000 |
| Sit correlation with Goals | 0.425806 | 0.4 | 0.018 |
| Sit correlation with Symptoms | 0.561206 | 0.6 | 0.002 |
| Sit correlation with Pain | 0.571441 | 0.6 | 0.002 |
| Sit correlation with Energy | 0.324682 | 0.3 | 0.072 |
| Sit correlation with Stiffness | 0.605892 | 0.6 | 0.001 |
| Sit correlation with Sleep | 0.142051 | 0.1 | 0.442 |
| Sit correlation with Depression | 0.466926 | 0.5 | 0.011 |
| Sit correlation with Memory | 0.266239 | 0.3 | 0.140 |
| Sit correlation with Anxiety | 0.293174 | 0.3 | 0.105 |
| Sit correlation with Tenderness | 0.297109 | 0.3 | 0.103 |
| Sit correlation with Balance | 0.369223 | 0.4 | 0.046 |
| Sit correlation with Environment | 0.401394 | 0.4 | 0.027 |
| Shop correlation with Goals | 0.602414 | 0.6 | 0.001 |
| Shop correlation with Symptoms | 0.568546 | 0.6 | 0.002 |
| Shop correlation with Pain | 0.633287 | 0.6 | 0.001 |
| Shop correlation with Energy | 0.384015 | 0.4 | 0.038 |
| Shop correlation with Stiffness | 0.567775 | 0.6 | 0.002 |
| Shop correlation with Sleep | 0.287735 | 0.3 | 0.129 |
| Shop correlation with Depression | 0.346023 | 0.3 | 0.064 |
| Shop correlation with Memory | 0.498545 | 0.5 | 0.007 |
| Shop correlation with Anxiety | 0.297406 | 0.3 | 0.108 |
| Shop correlation with Tenderness | 0.287698 | 0.3 | 0.124 |
| Shop correlation with Balance | 0.560411 | 0.6 | 0.003 |
| Shop correlation with Environment | 0.307212 | 0.3 | 0.099 |
| Goals correlation with Symptoms | 0.680049 | 0.7 | 0.000 |
| Goals correlation with Pain | 0.727288 | 0.7 | 0.000 |
| Goals correlation with Energy | 0.538972 | 0.5 | 0.003 |
| Goals correlation with Stiffness | 0.553772 | 0.6 | 0.002 |
| Goals correlation with Sleep | 0.473503 | 0.5 | 0.011 |
| Goals correlation with Depression | 0.266815 | 0.3 | 0.144 |
| Goals correlation with Memory | 0.311695 | 0.3 | 0.084 |
| Goals correlation with Anxiety | 0.234539 | 0.2 | 0.195 |
| Goals correlation with Tenderness | 0.343326 | 0.3 | 0.060 |
| Goals correlation with Balance | 0.376060 | 0.4 | 0.043 |
| Goals correlation with Environment | 0.144765 | 0.1 | 0.427 |
| Symptoms correlation with Pain | 0.817389 | 0.8 | 0.000 |
| Symptoms correlation with Energy | 0.651253 | 0.7 | 0.000 |
| Symptoms correlation with Stiffness | 0.360032 | 0.4 | 0.050 |
| Symptoms correlation with Sleep | 0.470726 | 0.5 | 0.012 |
| Symptoms correlation with Depression | 0.409578 | 0.4 | 0.027 |
| Symptoms correlation with Memory | 0.465180 | 0.5 | 0.011 |
| Symptoms correlation with Anxiety | 0.380034 | 0.4 | 0.039 |
| Symptoms correlation with Tenderness | 0.459459 | 0.5 | 0.013 |
| Symptoms correlation with Balance | 0.510802 | 0.5 | 0.007 |
| Symptoms correlation with Environment | 0.417511 | 0.4 | 0.024 |
| Pain correlation with Energy | 0.562092 | 0.6 | 0.002 |
| Pain correlation with Stiffness | 0.452461 | 0.5 | 0.013 |
| Pain correlation with Sleep | 0.510630 | 0.5 | 0.006 |
| Pain correlation with Depression | 0.443113 | 0.4 | 0.016 |
| Pain correlation with Memory | 0.411765 | 0.4 | 0.023 |
| Pain correlation with Anxiety | 0.400002 | 0.4 | 0.028 |
| Pain correlation with Tenderness | 0.365499 | 0.4 | 0.046 |
| Pain correlation with Balance | 0.392274 | 0.4 | 0.035 |
| Pain correlation with Environment | 0.251678 | 0.3 | 0.169 |
| Energy correlation with Stiffness | 0.288526 | 0.3 | 0.112 |
| Energy correlation with Sleep | 0.510630 | 0.5 | 0.006 |
| Energy correlation with Depression | 0.416257 | 0.4 | 0.023 |
| Energy correlation with Memory | 0.503268 | 0.5 | 0.005 |
| Energy correlation with Anxiety | 0.419674 | 0.4 | 0.021 |
| Energy correlation with Tenderness | 0.159490 | 0.2 | 0.384 |
| Energy correlation with Balance | 0.385392 | 0.4 | 0.038 |
| Energy correlation with Environment | 0.304662 | 0.3 | 0.095 |
| Stiffness correlation with Sleep | 0.136615 | 0.1 | 0.463 |
| Stiffness correlation with Depression | 0.303115 | 0.3 | 0.099 |
| Stiffness correlation with Memory | 0.078689 | 0.1 | 0.665 |
| Stiffness correlation with Anxiety | 0.118421 | 0.1 | 0.516 |
| Stiffness correlation with Tenderness | 0.313361 | 0.3 | 0.088 |
| Stiffness correlation with Balance | 0.138092 | 0.1 | 0.460 |
| Stiffness correlation with Environment | 0.179411 | 0.2 | 0.328 |
| Sleep correlation with Depression | 0.293735 | 0.3 | 0.118 |
| Sleep correlation with Memory | 0.299569 | 0.3 | 0.107 |
| Sleep correlation with Anxiety | 0.396184 | 0.4 | 0.033 |
| Sleep correlation with Tenderness | 0.083069 | 0.1 | 0.658 |
| Sleep correlation with Balance | 0.229404 | 0.2 | 0.230 |
| Sleep correlation with Environment | 0.055193 | 0.1 | 0.768 |
| Depression correlation with Memory | 0.416257 | 0.4 | 0.023 |
| Depression correlation with Anxiety | 0.734211 | 0.7 | 0.000 |
| Depression correlation with Tenderness | 0.157005 | 0.2 | 0.397 |
| Depression correlation with Balance | 0.219148 | 0.2 | 0.245 |
| Depression correlation with Environment | 0.380988 | 0.4 | 0.039 |
| Memory correlation with Anxiety | 0.386887 | 0.4 | 0.033 |
| Memory correlation with Tenderness | 0.166136 | 0.2 | 0.364 |
| Memory correlation with Balance | 0.536796 | 0.5 | 0.004 |
| Memory correlation with Environment | 0.344401 | 0.3 | 0.059 |
| Anxiety correlation with Tenderness | -0.026669 | 0.0 | 0.885 |
| Anxiety correlation with Balance | 0.227852 | 0.2 | 0.222 |
| Anxiety correlation with Environment | 0.292373 | 0.3 | 0.110 |
| Tenderness correlation with Balance | 0.104959 | 0.1 | 0.578 |
| Tenderness correlation with Environment | 0.457915 | 0.5 | 0.013 |
| Balance correlation with Environment | 0.299872 | 0.3 | 0.111 |

Table S3 shows the correlation coefficient and associated *p*-value (rounded to 1 and 3 decimals, respectively, for display purposes) for each question with the remaining questions, as summarized in Fig. 1 in the main paper. We made use of Kendall’s tau correlation coefficient as it is a non-parametric method better suited to small groups with multiple tied values [4]. Coefficients range between –1 and 1 are considered practically visible if above 0.3 or below –0.3 (indicating an inverse relationship) and as practically significant is above 0.5 or below –0.5. Since we did not have a truly randomised sample, we focus more on practical significance than statistical significance; however, we also report the associated *p*-values indicating statistically significant associations if less than 0.05.

# S4: Normalized data of the original NMR spectral bins

Division of the original ^1^H NMR spectrum of each analysed urine sample into 0.02 ppm equal-sized bins, between 0.5 ppm and 10 ppm, yielded 468 bins with spectral data, excluding the region of the water peak (4.66–4.90 ppm). To account for dilution differences common to urine samples, each spectral bin was made relative to the CH_3_ singlet of creatinine at 3.13 ppm. The raw, normalized spectral data matrix of every analysed sample in this study is given as an electronic file (Excel format) in Table S4.

**Table S4:** Raw NMR spectral data (Excel format) normalized relative to the CH_3_ singlet of creatinine at 3.13 ppm, given as an electronic file (See Additional File S2 – Raw data matrix) attached online as part of the SI.

# S5: Outlier identification

Outliers were detected using equidistant binning data to identify cases with undue influences on the predictor models. This was done by assessing the principal components analysis (PCA) score plots for each of the experimental groups as shown in Fig S2(e–h). Cases presenting outside of the confidence interval boundary are classified as outliers. Similarly, a Hotelling’s T^2^ (Fig S2 a–d) analysis was also applied to the same data for supplementary outlier detection. Cases presenting above the solid horizontal line are classified as outliers. These two methods were then used in conjunction to identify outliers. Cases identified by either method were excluded from further analysis.


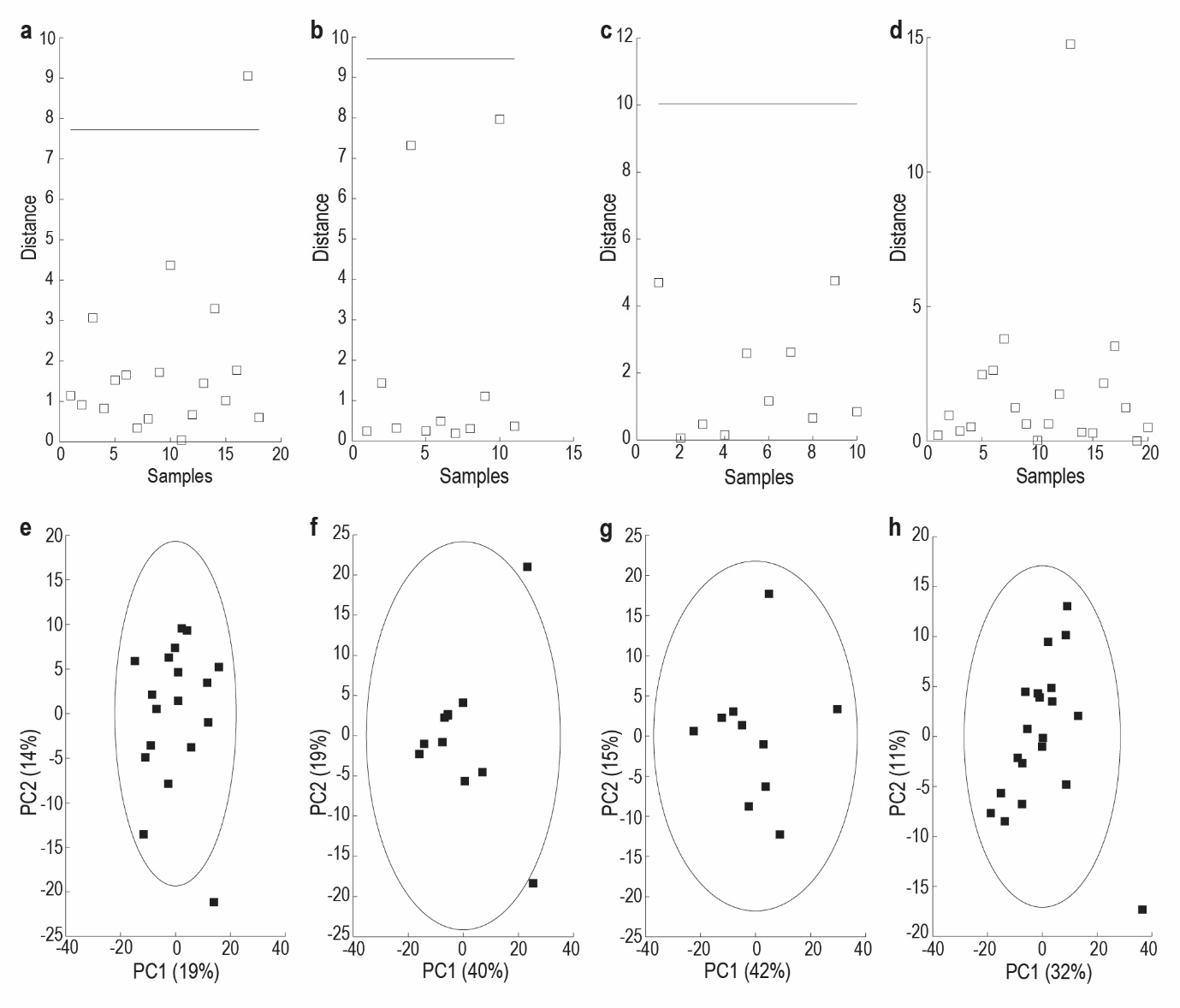


Figure S2: Outlier detection by means of a Hotelling’s T^2^ distance plot (a–d) and PCA scores plot (e–h) for each of the four experimental groups. Cases appearing above the red line (Hotelling’s T^2^ plot) or outside the blue confidence interval boundary (PCA scores plot) were earmarked as outliers.

# S6: PCA Analysis

Principal component analysis (PCA) was also performed on the entire scaled dataset, including all four groups. This provides a more holistic view of the variation in the data relative to the groups.


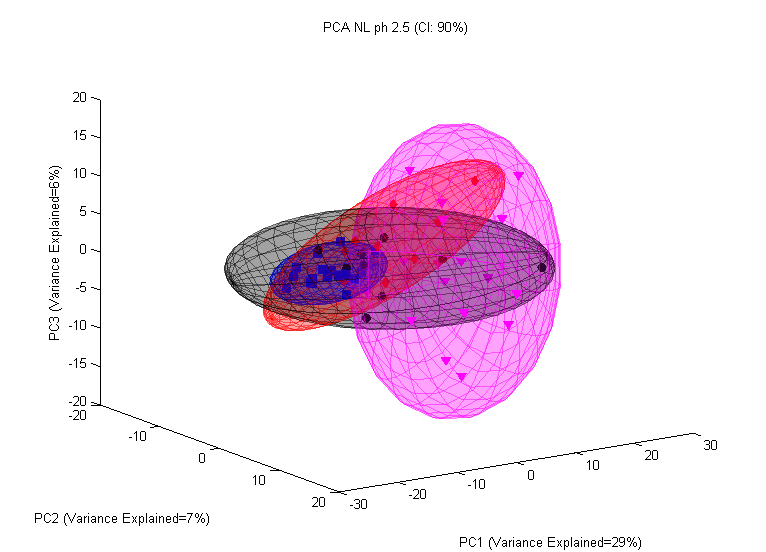


Figure S3: PCA of FMS (magenta); CF (black); CN (blue) and CO (red) groups

# S7: *N*-acetyl aspartic acid (NAA) verification

## Overview

Peak 9 in Fig. 2 of the main article was initially labelled as *N-*acetylaspatic acid. However, this peak is a characteristic moiety of most *N*-acetyl compounds and thus this result necessitated more detailed NMR analyses, by means of two-dimensional (2D) NMR, for verification. Of these 2D analyses we opted for correlation spectroscopy (COSY) and J-resolved spectroscopy (JRES).

## Reagents

The reagents used for this analysis were three of the study’s experimental subjects’ urine, namely, two Pre patient samples (Pre 1 and Pre 2) and one control sample from the CN group. A 1 mM sample of pure NAA compound (Sigma-Aldrich) was prepared in MilliQ-water. The samples were then spiked with this pure compound for analysis.

## Sample preparation and analysis

The two patients’ urine samples and the pure NAA compound were prepared as per the protocol described in the main article. Only the CN sample was spiked with the pure NAA compound as the aim of this analysis was to identify and verify NAA in the patient samples; we also required a urine control to see where the spiked NAA peak would present in urine. As such, the preparation protocol was adjusted for the CN sample, to take into account the addition of the pure NAA, by adding 630 µl of the centrifuged urine supernatant, 70 µl pure 1 mM NAA compound and 70 µl of internal standard (IS), which was TSP. the samples were adjusted to pH 2.5. All four samples were then analysed on ^1^H NMR, COSY and JRES. For the purposes of verification, the ^1^H NMR analysis was done at 512 scans to reduce the noise peaks to a minimum. The results of this analysis are shown in Fig. S4.

## Results and discussion

Fig. S4 shows the 1H NMR overlay of the four analysed samples. Red indicates the pure NAA compound, green indicates the spiked CN urine sample, and orange and light blue indicate the non-spiked Pre samples. The circled areas show the regions of interest where the pure NAA compound and NAA+ urine (spiked CN urine) peaks appear. Within the blue circled area in Fig. S4A, we can see that all the samples present with a singlet peak, which is characteristic of any *N*-acetyl compound, as also identified in the JRES (Fig. S4B) analysis shown in the corresponding blue area. In the yellow circled area we can see that all samples peak in this area, with the red sample being the highest. This peak depicts the characteristic multiplet peak of NAA. In the JRES picture we see that only the red and green samples present with this multiplet peak as the peak patterns correlate. All other peaks presenting in this area of the other samples can be concluded as not including the characteristic NAA peak as the peak patterns do not match those of the CN (green) and pure NAA (red) peaks.


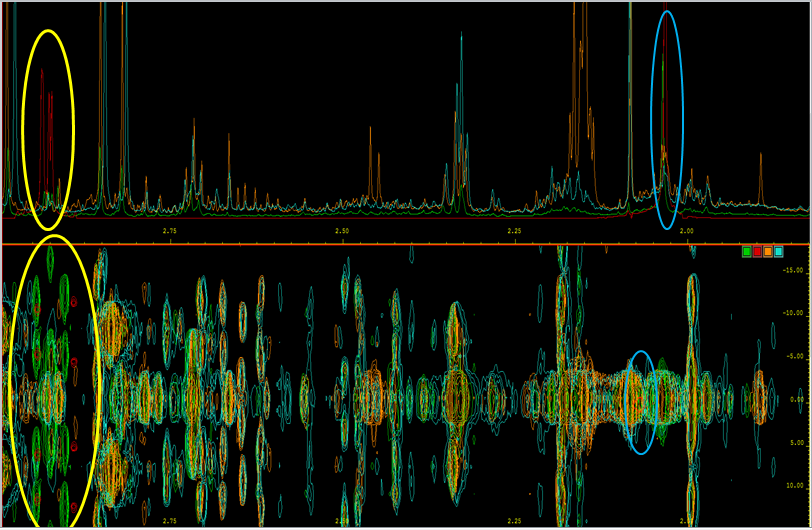


**B**

**A**


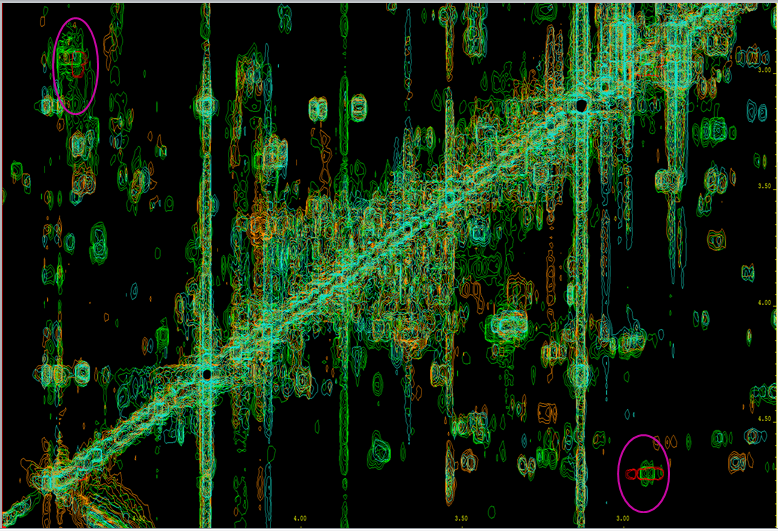


**C**

Figure S4: ^1^H NMR overlay of three urine samples, namely, two PRE patient samples (orange and blue) and a CN control group sample (green) spiked with pure N-acetyl aspartic acid. Red indicates the pure NAA compound dissolved in MilliQ water. Picture A shows the one-dimensional NMR analysis whereas B and C show the two-dimensional JRES and COSY analyses, respectively. The circled areas show the regions of interest for the verification of the presence of NAA.

# References:

1. Burckhardt CS, Clark SR, Bennett RM. The Fibromyalgia Impact Questionnaire: development and validation. J Rheumatol. 1991;18:728 733.
2. The overview of the FIQR and SIQR. <http://fiqrinfo.ipage.com/index.html>. Accessed on 22 January 2016.
3. Bennett RM, Friend R, Jones KD, Ward R, Han BK, Ross RL. The Revised Fibromyalgia Impact Questionnaire (FIQR): validation and psychometric properties. Arthritis Res Ther. 2009; 11(5):415.

Field, AP. Discovering statistics using IBM SPSS Statistics: and sex and drugs and rock 'n' roll. 3^rd^ ed. London: Sage Publications; 2009.
